# Identification and Functional Assessment of the First Placental Adhesin of *Treponema pallidum* That May Play Critical Role in Congenital Syphilis

**DOI:** 10.3389/fmicb.2020.621654

**Published:** 2020-12-21

**Authors:** Shekerah Primus, Sandra C. Rocha, Lorenzo Giacani, Nikhat Parveen

**Affiliations:** ^1^Department of Microbiology, Biochemistry and Molecular Genetics, Rutgers New Jersey Medical School, Newark, NJ, United States; ^2^Department of Medicine, Division of Allergy and Infectious Diseases, University of Washington, Seattle, WA, United States; ^3^Department of Global Health, University of Washington, Seattle, WA, United States

**Keywords:** *Treponema pallidum* subspecies *pallidum*, Tp0954, congenital syphilis, placenta binding adhesin, surrogate system, *Borrelia burgdorferi*, heterologous expression system

## Abstract

Syphilis is a global, re-emerging sexually transmitted infection and congenital syphilis remains a major cause of adverse pregnancy outcomes due to bacterial infection in developing nations with a high rate of fetus loss. The molecular mechanisms involved in pathogenesis of the causative agent, *Treponema pallidum* subsp. *pallidum* remain poorly understood due to the difficulties of working with this pathogen, including the inability to grow it in pure culture. To reduce the spread of syphilis, we must first increase our knowledge of the virulence factors of *T. pallidum* and their contribution to syphilis manifestations. Tp0954 was predicted to be a surface lipoprotein of *T. pallidum*. Therefore, we experimentally demonstrated that Tp0954 is indeed a surface protein and further investigated its role in mediating bacterial attachment to various mammalian host cells. We found that expression of Tp0954 in a poorly adherent, but physiologically related derivative strain of the Lyme disease causing spirochete *Borrelia burgdorferi* B314 strain promotes its binding to epithelial as well as non-epithelial cells including glioma and placental cell lines. We also found that Tp0954 expression facilitates binding of this strain to purified dermatan sulfate and heparin, and also that bacterial binding to mammalian cell lines is mediated by the presence of heparan sulfate and dermatan sulfate in the extracellular matrix of the specific cell lines. These results suggest that Tp0954 may be involved not only in initiating *T. pallidum* infection by colonizing skin epithelium, but it may also contribute to disseminated infection and colonization of distal tissues. Significantly, we found that Tp0954 promotes binding to the human placental choriocarcinoma BeWo cell line, which is of trophoblastic endocrine cell type, as well as human placental tissue sections, suggesting its role in placental colonization and possible contribution to transplacental transmission of *T. pallidum*. Altogether, these novel findings offer an important step toward unraveling syphilis pathogenesis, including placental colonization and *T. pallidum* vertical transmission from mother to fetus during pregnancy.

## Introduction

Syphilis is a chronic, systemic disease that is caused by the spirochete *Treponema pallidum* subspecies *pallidum* (*T. pallidum*). This pathogen is transmitted by sexual contact and vertically during pregnancy from mother to fetus. In recent years, syphilis has been re-emerging as a global health problem (Hook and Peeling, 2004). The most recent estimates by the World Health Organization reported the global burden of syphilis at ~6 million new cases per year among men and women of 15–49 years of age (WHO, 2012; Kojima and Klausner, 2018; Rowley et al., 2019). In the United States, the total number of syphilis cases reported in 2018 was the highest recorded in years, with a 71.4 and 185.3% increase in syphilis and congenital syphilis cases, respectively, compared to cases reported in the year 2014 (CDC, 2019).

Congenital diseases are the leading cause of infant morbidity and mortality. Several pathogens are transmitted vertically from mother to child during pregnancy including *Toxoplasma*, Other (syphilis, varicella-zoster syndrome, parvovovirus B19, hepatitis B), Rubella, Cytomegalovirus, and Herpes, i.e., TORCH infections. More recently, Zika virus transmission by congenital route has demonstrated tremendous harmful effects on fetal development (Ostrander and Bale, 2019). A few other pathogens, such as *Plasmodium falciparum* and *Trypanosoma cruzi* can also be transmitted through the vertical, transplacental route from mother to fetus (Carlier et al., 2012). Among these infectious diseases, the molecular basis of mother to child transmission that results in congenital syphilis remains almost completely unexplored. Congenital syphilis is a very serious infectious disease that can result in stillbirth, neonatal death, and debilitating disease in surviving infants (Cooper and Sanchez, 2018). The striking increase in congenital syphilis cases is of particular concern, as these infections are a leading cause of loss of fetus in developing nations, and the 2nd leading cause of preventable stillbirths worldwide. Treatment of infected women during pregnancy can significantly lower the incidence of congenital syphilis; however, the lack of proper prenatal care and access to testing and treatment remains a problem for women in many underprivileged settings. It is therefore essential to reveal the mechanisms used by *T. pallidum* to colonize and cross placental syncytiotrophoblast cell barrier to infect the fetus. Such an understanding will ultimately lead to the development of an effective vaccine to prevent congenital transmission of this pathogen similar to that undergoing testing for malaria (Fried and Duffy, 2015).

Although treatment of patients with Benzathine G is highly effective and cure of patients infected with *T. pallidum* is very successful, the lack of proper prenatal care in many resource limiting regions of the world continues to result in high number of cases of congenital syphilis in many countries. Development and employment of a vaccine against syphilis, particularly using virulence factors involved in placental colonization, is the best option to restrict vertical transmission of *T. pallidum* from mother to child for resources limiting regions of the world. A successful vaccine can then be offered to adolescent women to prevent this devastating disease throughout their fertile age. Surface-exposed lipoproteins of exclusively extracellular pathogen *T. pallidum* are essential for initial infection, dissemination to distal tissues, and chronic pathogenesis. Study of syphilis has been dependent on propagation of *T. pallidum* in rabbit testes and examination of orchitic and skin lesions in the rabbit model of infection (Morgan et al., 2002; Pereira et al., 2020). Recovery of these spirochetes often damages their fragile outer membrane making it difficult to unambiguously determine the localization of various proteins at the subcellular level of *T. pallidum*, preventing a consensus among researchers on whether a protein is located on the spirochete surface or periplasmic space.

Genome sequence of *T. pallidum* identified 22 putative lipoproteins (Fraser et al., 1998). Based upon previously published reports and the SpLip algorithm to predict lipoproteins, we had listed 40 potential *T. pallidum* lipoproteins (Chan et al., 2016); however, characterization of these molecules remains slow for several reasons. First, while the localization of many *T. pallidum* lipoproteins still remains unconfirmed, studies have shown that many of them are not surface-exposed; instead, they are located in the periplasmic space of the bacteria. Lack of surface-exposed antigens makes *T. pallidum* rather inert and likely increases better survival of this pathogen by evading the immune response (Radolf et al., 2016) during infection. It is still difficult for scientists to determine which lipoproteins of this extracellular pathogen are essential for host-bacterial interactions. Edmondson and coworkers recently reported (Edmondson et al., 2018) their success in co-culture of different strains of *T. pallidum* with rabbit epithelial Sf1Ep cells continuously for a long period of time. Inability to maintain *T. pallidum* in pure culture has slowed development of its genetic manipulation strategies, thus hindering direct confirmation of the roles of its virulence factors. To fully understand the cause of syphilis manifestations and develop effective vaccine(s) against this disease requires that we identify and characterize important surface proteins and lipoproteins of *T. pallidum*. Thus, it is essential to assess function of surface proteins/lipoproteins of *T. pallidum* in a systematic manner.

To overcome limitations that prevent studies with physiologically active *T. pallidum* directly, we have developed a heterologous expression system that allows us to assess the subcellular localization of *T. pallidum* proteins and determine their functions by using the Lyme disease causing bacterium, *Borrelia burgdorferi* (*B. burgdorferi*) as a surrogate expression system. *B. burgdorferi* is also an extracellular spirochetal pathogen like *T. pallidum* and colonizes several overlapping organs leading to multisystemic Lyme disease similar to syphilis that affects skin, joints, brain and heart; however, it can be propagated *in vitro* in pure culture and genetic manipulation protocols for this spirochete are now well-established. Furthermore, these two spirochetal bacteria have significant structural and physiological similarities with each possessing unique and distinct virulence factors (Subramanian et al., 2000). We have previously used the non-infectious, poorly adherent *B. burgdorferi* strain B314 (Sadziene et al., 1993) in our studies to express and study the function of *T*. *pallidum* putative surface proteins and evaluate their potential as vaccine candidates (Chan et al., 2016; Djokic et al., 2019; Parveen et al., 2019). There are no cases of congenital Lyme disease reported until now. Thus, *B. burgdorferi* is an ideal expression system to study *T. pallidum* virulence factors, especially those involved in congenital transmission such that the use of infectious *B. burgdorferi* surrogate may be possible to study syphilis pathogenesis in the selected animal models in the future.

We are interested in investigating surface proteins/lipoproteins and potential virulence factors of *T. pallidum*, determine their function, evaluate them as candidates for both, specific diagnosis of syphilis and as protective antigens for vaccine development. In this study, we examined a putative surface *T. pallidum* lipoprotein, Tp0954 by employing our gain-in-function approach and evaluated its function using *in vitro* strategies. We confirmed expression of Tp0954 in *B. burgdorferi* B314 strain and found that Tp0954 expression confers poorly adherent B314 strain ability to bind to epithelial and non-epithelial mammalian cell lines by recognition of heparan sulfate and dermatan sulfate. Tp0954 also facilitates binding to cells derived from placenta as well as placental tissue. This is the first study to demonstrate the molecular basis of adherence of *T. pallidum* to placental cell line and even to human placental tissue. Overall, this work advances our understanding of *T. pallidum* pathogenesis and provides insight into its placental colonization that could lead to developing a vaccine to potentially inhibit its transplacental transmission, and thus prevent congenital syphilis in the future.

## Materials and Methods

### Ethics Statement

Polyclonal antibodies against recombinant Tp0954 were produced in BALB/c mice using the previously described protocol (Parveen and Leong, 2000). All mouse experiments were performed in accordance with all provisions of the Animal Welfare Act, the Guide for the Care and Use of Laboratory Animals, and the PHS Policy on Humane Care and Use of Laboratory Animals. These experiments were conducted under the protocol number 14011D0617 approved by the Rutgers Biomedical and Health Sciences IACUC.

Samples were collected from the placenta after full-term normal birth at University Hospital of the New Jersey Medical School (NJMS), Rutgers University at Newark, New Jersey under approved IRB protocol of Dr. Abraham Aviv, who initiated his placental study at Rutgers University. All mothers for this component of his work signed a written informed consent. Dr. Aviv provided frozen and fixed placental tissues to Dr. Herbig Utz of NJMS who generously provided left-over frozen tissue pieces to Parveen's laboratory for this study after obtaining Dr. Aviv's consent.

### Heterologous Expression of *T. pallidum* Tp0954 in *Borrelia burgdorferi*

Tp0954 is listed as a conserved tetratricopeptide (TPR) structural domain-containing protein on genome sequence of *T. pallidum* Nichols strain (Fraser et al., 1998). Among various TPR domain containing proteins listed in *T. pallidum* genome bank, based upon our bioinformatics analysis Tp0954 is predicted to be both, (i) a lipoprotein, and (ii) potentially localized on the surface of spirochetes (Supplementary Figure 1). Therefore, Tp0954 coding region was codon optimized for expression in *B. burgdorferi* using the GenScript codon optimization service (GenScript, Inc., NJ) and clone was provided in pUC57 vector. The codon optimized Tp0954 ORF was synthesized downstream of the *B. burgdorferi* Outer surface protein C (OspC) (Sadziene et al., 1993) encoding gene promoter. The synthesized construct was digested out of the pUC57 vector using Sal1 and Sph1and cloned into pJSB175 shuttle vector. The plasmid clone obtained by such ligation was used to transform *E. coli* chemically competent Top10 strain using standard techniques. Positive clones were identified by restriction digestion and confirmed by sequencing. The shuttle vector pJSB175 containing Tp0954 was used to transform electrocompetent *B. burgdorferi* B314 strain as previously described (Chan et al., 2016). Colonies of transformed B314 were grown in *B. burgdorferi* broth medium, plasmid DNA extracted and sequenced to confirm *B*. *burgdorferi* B314 contained pJSB175 with optimized *tp0954* gene and labeled as B314(pTp-Bb). Transformation of B314 strain containing the empty pJSB175 vector control, B314(V), was described previously (Chan et al., 2016).

### Reverse Transcription Polymerase Chain Reaction (RT-PCR)

Tp0954-expressing and control *B. burgdorferi* broth cultures were centrifuged and the pellet washed three times with 1X PBS. Total RNA was then isolated using TRIzol reagent (Thermo Scientific), followed by RNA purification using the RNeasy kit (Qiagen) according to the manufacturers' instructions. Contaminating DNA was removed from RNA using 1U/μg RQ1 RNase-free DNase (Promega) following manufacturer's protocol. The purified RNA was then used for cDNA synthesis and PCR using the Superscript III one-step RT-PCR system (Invitrogen). Primers TGGAACTCTTTTTAAAGTTAGTGT and AGAAGGAGCTCTTGCAGATTCAA were used to amplify Tp0954, and luciferase gene was amplified as internal control.

### *B. burgdorferi* Surrogate Strain and Culture Conditions

The high passage, non-infectious, poorly adherent *B*. *burgdorferi* B314 strain (Chan et al., 2016) was used for all experiments. All strains were cultured in 1X Barbour-Stoenner-Kelly (BSK II) medium containing 6% Rabbit Serum at 33°C.

### Mammalian Cell Culture

Mammalian cell lines: Vero (CCL81) green monkey kidney epithelial cells, 293 human embryonic kidney (HEK293) epithelial cells (CRL1573), EA.hy926 (CRL2922) human endothelial cells, BeWo (CCL98) human placental cells, and C6 (CCL107) rat glial cells, were obtained from the American Type Culture Collection (ATCC) or brought from Dr. John Leong's laboratory at the University of Massachusetts Medical School in Worcester, Massachusetts and cultured in respective media at 37°C and 5% CO_2_ as previously described (Chan et al., 2016).

### Purification of Recombinant Polyhistidine-Tagged Tp0954

A liter culture of BL21(pLysS) strain of *E. coli* containing *tp0954* gene cloned in pET30a plasmid was grown at 37°C with shaking until absorbance at 600 nm reached ~0.6, i.e., culture reached mid-logarithmic phase of growth. Expression of polyhistidine-tagged full size Tp0954 protein or that containing the N-terminal 281 amino acids without signal peptide was induced using 1 mM IPTG by incubation at 37°C overnight without shaking. The next day, supernatant was discarded after centrifugation and bacterial pellet resuspended in PBS containing 1 mg/ml lysozyme and incubated at 37°C for 30 min. Bacterial culture was lysed by sonication in the presence of proteinase inhibitor cocktail (Sigma-Aldrich Inc. MO) while placed on ice. The lysed bacterial pellet was removed by centrifugation, supernatant filtered through 0.22 μ syringe filter and recombinant Tp0954 purified following the manufacturer's kit and protocol (Novagen, WI).

### Production of Polyclonal Antibodies in Mice Against the Recombinant Tp0954

Recombinant purified Tp0954 was mixed with an equal volume of Complete Freund's Adjuvant (CFA) at 1:1 ratio and emulsion prepared by repeated vortexing in cold. Each mouse was injected with 0.1 ml of emulsion. This step was repeated to provide two booster doses at 2 weeks interval using Incomplete Freund's adjuvant instead of CFA for emulsion preparation. Mice were bled a week after the second booster dose by retro-orbital bleeding and titer of sera determined by Western blotting using recombinant Tp0954 protein as antigen. After a week's rest following the bleeding, mice were given a third and final boost with Incomplete Freund's Adjuvant emulsion of the recombinant protein. One week after this injection, blood was collected by cardiac puncture and mice were euthanized. Sera were separated by centrifugation and stored at −20°C until use.

### Indirect Immunofluorescence Assay (IFA)

**Figure 1:**
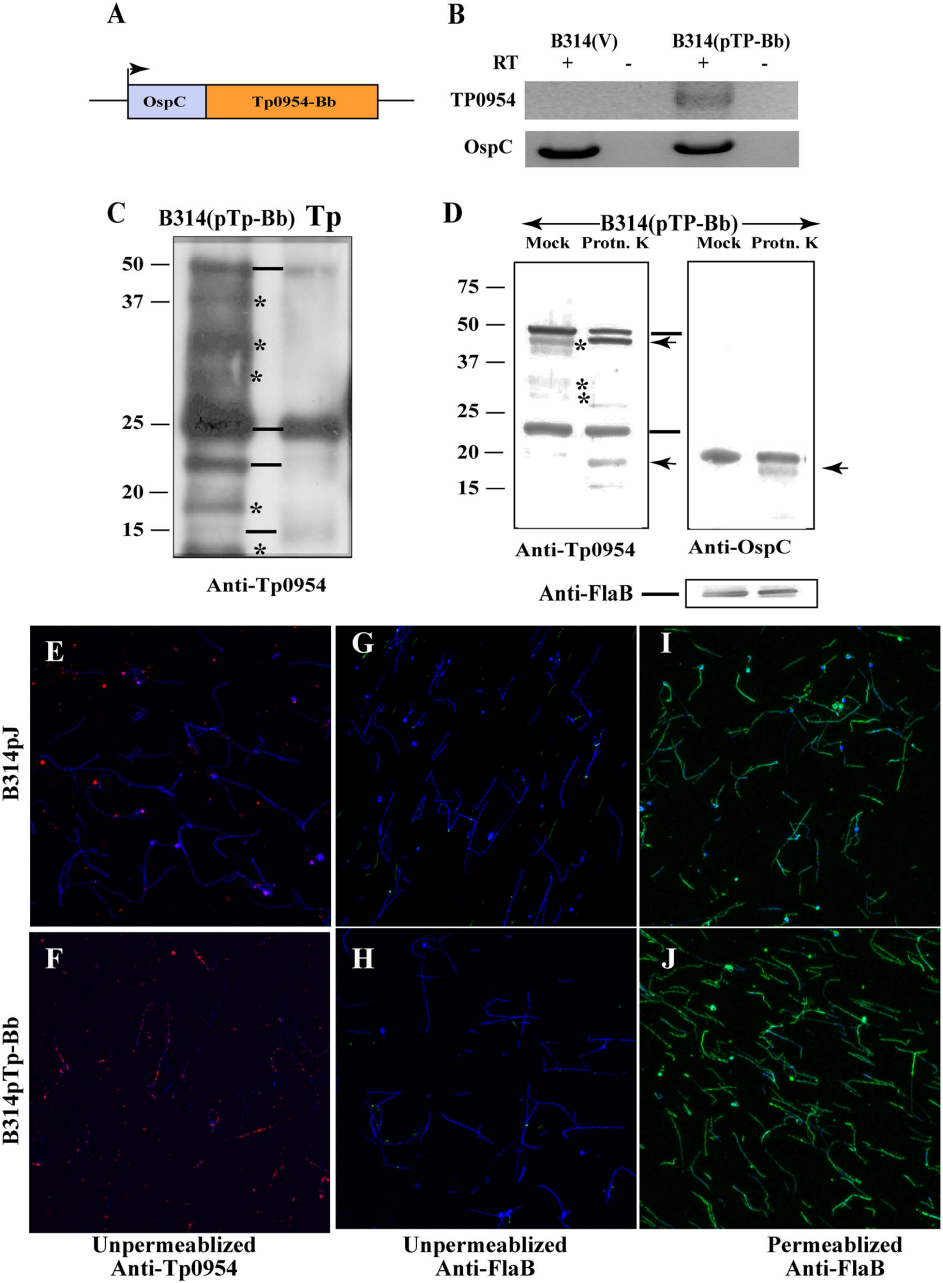
Expression of Tp0954 in *B. burgdorferi* B314 strain and detection on the surface of transformed spirochetes. **(A)** Schematic of the construct expressing the Tp0954 coding region driven by the *B. burgdorferi ospC* promoter (pTp-Bb). **(B)** Tp0954 expression in B314(pTp-Bb) and not vector transformed B314(V) strain was detected by RT-PCR using Tp0954 primers. Positive control RT-PCR is also shown in which primers were used to amplify the *ospC* gene of *B. burgdorferi*. RT-PCR products were absent when reverse transcriptase was not included in the reaction indicting specificity of RT-PCR. **(C)** Detection of three Tp0954 protein bands (marked by horizontal lines) in *T. pallidum* (Tp) Nichols strain as well as Tp0954 transformed B314 strain, B314(pTp-Bb) using antibodies against 281 N-terminal amino acids of mature Tp0954 protein (anti-Tp0954N) suggest differential post-translational processing of full size Tp0954 protein of ~50 kD size. *Non-specific binding with cross reactivity of antibodies with unrelated B314 proteins. **(D)** Limited proteinase K treatment of intact spirochetes digested only surface Tp0954 versions and *B. burgdorferi* OspC protein but not periplasmic flagellar protein. **(E,F)** IFA using anti-Tp0954N primary antibodies followed by TRITC-labeled secondary antibodies using unpermeabilized spirochetes confirmed the surface localization of Tp0954 only in Tp0954 transformed B314 and not in the control, B314(V), strain. **(G,H)** The lack of staining of flagella in unpermeabilized spirochetes by anti-FlaB monoclonal antibodies treatment followed by anti-mouse Alexa fluor 488 antibodies indicate that integrity of outer membrane of B314 was maintained during IFA procedure. **(I,J)** Staining of *B. burgdorferi* flagella after permeabilization with methanol indicates the specific reactivity of anti-FlaB antibodies. All spirochetes present in each microscopic field are detected by co-labeling with DNA stain, DAPI and superimposed images are shown in **(E–J)**. Bar indicates 20 μ size.

After centrifugation and washing of transformed *B. burgdorferi* culture pellet with PBS containing 0.2% BSA (PBS/0.2% BSA), spirochetes were centrifuged on the 12 mm coverglass placed in 24 well tissue culture plate. After removing supernatant, bacteria were fixed using 3% paraformaldehyde made in PBS and filtered through 0.22μ filter for 1 h and the solution was then removed. After blocking with PBS containing 5% BSA and 5% heat inactivated goat serum, *B. burgdorferi* were labeled with primary antibodies generated against 281 amino acids of N-terminal of mature Tp0954 in BALB/c at 1:100 dilution or monoclonal antibodies against FlaB protein (1:50 dilution) followed by 1:600 dilution of secondary anti-mouse antibodies conjugated to TRITC for Tp0954 and 1:200 dilution of Alexa fluor 488 conjugated antibodies (Life Technologies-Molecular Probes, MA) for FlaB using the previously described protocol (Chan et al., 2016). Simultaneous staining of DNA with 4′,6-diamidino-2-phenylindole (DAPI) allowed us to detect all bacteria present in any given microscopic field of view. Permeabilization of *B. burgdorferi* with cold methanol for 20 min followed by labeling with antibodies determined localization of proteins in the periplasmic region. The lack of staining by anti-FlaB mouse antibodies in unpermeabilized spirochetes ascertained that the integrity of the outer membrane was maintained in these *B. burgdorferi*. Staining with anti-FlaB antibodies after permeabilization followed by secondary antibodies were used to detect periplasmic flagella and confirm reactivity of the antibodies. Spirochetes images were captured using Confocal Z-stack using a Nikon-A1R confocal microscope equipped with a 60x Plan Apo VC oil immersion objective. Superimposed images with DAPI and specific antibody stained bacteria are shown in the Figure 1.

### *In vitro* Propagation of *T. pallidum* Strain and IFA

We adapted the *T. pallidum* culture system described by Edmondson et al. (2018) to perform IFA analysis on *T. pallidum* grown *in vitro*. More specifically, after passaging treponemes in six-well culture plates (Corning Inc, Corning, NY), spirochetes were sub-cultured into 8-well chamber slides (Lab-Tek New Holland, PA). Briefly, the day before treponemal inoculation, slide chambers were seeded with 6 × 10^3^ rabbit Sf1Ep cells (kindly provided by Dr. Diane Edmondson and Dr. Steven Norris, UTHealth) per well in 300 μl of culture media. The slides were then incubated overnight in a 5% CO_2_ atmosphere within a HeraCell 150 incubator (Thermo Fisher Scientific) to facilitate cellular adhesion to the slide surface. On the same day, TpCM-2 media was prepared according to the Edmondson et al. protocol (Edmondson et al., 2018). The TpCM-2 media was equilibrated overnight at 34°C in a microaerophilic environment consisting of 1.5% O_2_, 3.5% CO_2_ and 95% N_2_ supplied in a tri-gas incubator (Thermo Fisher Scientific). On the following day, cell culture media was removed from the chambers, and cells were rinsed with equilibrated TpCM-2 media. Subsequently, each well was filled with 300 μl of equilibrated TpCM-2 media and the plate was transferred to the incubator for equilibration in the microaerophilic atmosphere for 3 h. To prepare the treponemal inoculum for the chambers, the Sf1Ep cells inoculated the previous week with *T. pallidum* were treated with trypsin to allow the release of spirochetes and their enumeration. Treponemes were counted using dark field microscopy on a Leica DM2500 LED microscope (Leica, Wetzlar, Germany) and diluted in TpCM-2 to 2.0 × 10^4^ *T. pallidum* cells/ml. We obtained a treponemal inoculum of 10^5^ cells in a total of 300 μl, which were added to each well of chamber slide. Treponemes were allowed to grow in the tri-gas incubator for 3 days, following which the media was removed and cells fixed with 3% buffered paraformaldehyde. A protocol similar to the one described above for *B. burgdorferi* IFA was used for *in vitro* co-cultured *T. pallidum* SS14 strain. In these IFA experiments, treatment with anti-Tp0954N antibodies were followed by detection and surface labeling of Tp0954 using anti-mouse TRITC-conjugated secondary antibody. Rabbit antibodies generated against FlaA and obtained from Drs. Steven Norris and Diane Edmondson were used for flagellar staining before and after permeabilization of *T. pallidum*. In this case, an Alexa Fluor 488-labeled secondary antibody at 1:400 was used for detection.

### Radioactive Labeling of *B. burgdorferi* and Binding Assays

*B. burgdorferi* B314 strains expressing either empty vector or Tp0954 were radiolabeled with [^35^S] methionine-cysteine mixture as previously described (Chan et al., 2016). Binding assays to mammalian cells were conducted by adding radiolabeled bacterial cultures directly to washed monolayers of Vero, HEK293, EA.hy926, BeWo and C6 mammalian cells in 96-well plates. Binding assays to glycosaminoglycans (GAGs) were conducted by adding radiolabeled bacteria directly to the GAGs, chondroitin sulfate A (sigma: C9819), dermatan sulfate (sigma: C3788), chondroitin sulfate C (sigma: C4384) and heparin (sigma: H3393), which were coated onto 96-well plates (Parveen and Leong, 2000). To determine whether removing GAGs from the surface of mammalian cells would affect bacterial binding, monolayers were first treated with one of the following GAG lyases: chondroitinase ABC (sigma: C3667), and heparinase I, III (sigma: H3917) for 2 h at 37°C as previously described (Leong et al., 1998; Parveen et al., 1999), to degrade respective GAGs before performing the binding assay. Luciferase-dependent binding assays were performed using bioluminescent, non-radiolabeled bacteria as previously described (Chan et al., 2016). Each binding experiment was conducted 4–5 times with one representative experiment result for each assay presented here.

Frozen and fixed placental tissue sections were thawed and rehydrated with PBS for 30'at room temperature. Cultures of B314(V) and B314(pTp-Bb) were washed one time with PBS/0.2% BSA, resuspended in 1:2 mixture of BSK-H and 10 mM glucose + 10 mM HEPES (pH 7.0) + 50 mM NaCl and then were allowed to bind for 2 h at 37°C. Bioluminescence was measured after adding D-luciferin substrate for measuring luciferase activity in the bound spirochetes. For IFA, samples were blocked as described above for IFA of *B. burgdorferi* and after two PBS washings, mixture of FITC conjugated anti-*B. burgdorferi* antibodies and Alexa fluor 647 conjugated wheat germ agglutinin lectin were allowed to react for 1 h at 37°C to label spirochetes and placental cells, respectively. After three washings with PBS, coverglasses were used to cover sections using 1:1 PBS:glycerol mixture, sealed with nail polish and microscopic images captured as described above. Experiment was conducted four times and results of two representative experiments are presented here (**Figure 5** and Supplementary Figure 4).

## Results

### Selection of the *T. pallidum* Putative Surface Lipoprotein Tp0954 for Functional Studies

We decided to pursue investigation of Tp0954 in this study because using TMHMM and LipoP 1.0 programs, it was predicted to be a surface lipoprotein (Supplementary Figures 1A,B, respectively) and putative virulence factor. Based upon homology blast search of structures, Tp0954 is predicted to be a TPR structural domain-containing protein that possesses tandem alpha helices (Supplementary Figure 1C) (Altschul et al., 1997; Arnold et al., 2006; Benkert et al., 2011; Biasini et al., 2014). Alpha helical domains of this subfamily of proteins are known to be involved in protein-protein interactions (Blatch and Lassle, 1999). Several TPR domain-containing proteins, which can assemble in higher molecular weight structures with homologous or heterologous proteins, have also been implicated in virulence of other bacterial pathogens (Cerveny et al., 2013). Collectively, this information prompted us to determine localization of Tp0954 lipoprotein of *T. pallidum* and evaluate its function. Tp0954 was confirmed to be highly conserved among various strain of *T. pallidum* with 100% identity (Supplementary Figure 2A). Interestingly, the only other *Treponema* species where Tp0954 showed almost perfect identity was Treponema *paraluiscuniculi*, which was reported to have evolved from a *T. pallidum*-like ancestor and was then adapted to rabbits after loss of infectivity in humans (Smajs et al., 2011) (Supplementary Figure 2B).

### Expression of Tp0954 in *B. burgdorferi* B314 Strain

We first cloned *tp0954* gene along with its predicted promoter region into a shuttle vector pJSB175 that possesses codon-optimized luciferase gene under strong constitutive *flaB* promoter of *B. burgdorferi* (Blevins et al., 2007). We transformed *B. burgdorferi* B314 strain with this plasmid. B314 strain has lost most of endogenous plasmids of *B. burgdorferi* parental B31 strain and thus lacks various virulence factors and several adhesins involved in Lyme disease multisystemic manifestations (Chan et al., 2016). *B. burgdorferi* is physiologically and structurally related to *T. pallidum*, making it an ideal surrogate expression system for *T. pallidum* genes. We were unable to detect significant expression of cloned *tp0954* gene in the transformants of B314 that had original *T. pallidum* open reading frame (ORF) (data not shown). To improve the expression of Tp0954 protein for our studies, we synthesized a version of the *tp0954* ORF that was codon-optimized for expression in *B. burgdorf* eri (GenScript Biotech, NJ). Additionally, the inducible promoter of *B. burgdorferi* outer surface protein C (*ospC*) gene was used to drive Tp0954 expression (Figure 1A). The use of the *ospC* promoter was expected to improve transcription of *tp0954* gene under *B. burgdorferi* culture conditions. This construct was cloned into the pJSB175 vector and used to transform B314 strain. Successful expression of Tp0954 in the B314 strain was confirmed by RT-PCR. RNA expression was detected in the bacteria transformed with the *tp0954* construct, B314(pTP-Bb) only when reverse transcriptase was included in the PCR reaction mixture and was absent in control B314(V) strain containing the empty pJSB175 vector (Figure 1B). Expression of the luciferase gene (*luc*) was similar in Tp0954-expressing and control bacteria (data not shown).

Preference of degenerate codons for incorporation of the specific amino acids during translation of proteins differs in different organisms and is determined by the genome sequence of the organism. Codon-optimization of a gene for expression in a surrogate system allows improved expression of protein without affecting either protein sequence or its structure. By using known codon preference of *B. burgdorferi* to construct Tp0954 expressing ORF, we expected that increased transcription of *tp0954* gene we observed will also result in appreciable level of expression of this protein in B314 strain to allow us to conduct functional studies. To further confirm the expression of Tp0954 protein in B314 strain, we generated antibodies against full-size recombinant protein in mice. Due to extensive cross-reactivity of these antibodies with other B314 proteins (data not shown), we further generated antibodies against 281 amino acids at the N-terminal of the mature Tp0954 protein (without signal peptide) that is predicted to be on the spirochete surface (Supplementary Figure 1). We found that although these antibodies also recognize some B314 proteins non-specifically, we were able to detect expected size of Tp0954 protein (~50 kD) in this strain by Western blotting (Figure 1C). A comparable albeit slightly smaller band was detected in Nichols strain of *T. pallidum* (Tp) possibly due to different lipid moiety incorporated in *B. burgdorferi* and *T. pallidum* lipoproteins. Indeed, preference for lipids in lipoproteins of these two spirochete species have been shown to be different (Belisle et al., 1994). Interestingly, we detected multiple isoforms both in *T. pallidum* strain and when Tp0954 is expressed in B314 strain as we had shown for another lipoprotein, Tp0435 previously suggesting different post-translational processing and/or modifications (Chan et al., 2016).

### Tp0954 Is Displayed on the Surface of B314 Strain Spirochetes

We used two different and complementary approaches to show that Tp0954 is expressed on the surface of *B. burgdorferi*. First, we treated intact B314(pTP-Bb) spirochetes with proteinase K in a limited manner and resolved total proteins by SDS-PAGE after heat inactivation of enzyme (Figure 1D). Untreated control strain (Mock) was included for comparison. Digestion of two Figure Tp0954 protein isoforms was detected by Western blotting. These results were similar to that observed for OspC of *B. burgdorferi* included as a positive control. The lack of digestion of periplasmic FlaB protein (bottom) from the same preparation of treated proteins and detected from the same Western blot parallel lanes indicate that only surface proteins of the spirochetes were cleaved by limited proteinase K treatment.

In an alternative approach, Tp0954 protein expression in B314 was confirmed by indirect immunofluorescence assay (IFA) using antibodies against N-terminal end of Tp0954 followed by anti-mouse TRITC conjugated secondary antibodies. All spirochetes present in each microscopic field of view were detected by simultaneously staining linear chromosome with DNA stain, 4′,6-diamidino-2-phenylindole (DAPI). Our results show the expression of Tp0954 protein on intact, unpermeabilized B314(pTP-Bb) spirochetes in a punctuate manner which was not observed in the control B314(V) *B. burgdorferi* strain (compare red fluorescence in Figure 1F with that in Figure 1E) indicating that Tp0954 is exposed on the surface of this heterologous spirochete expression system. Interestingly, a few *B. burgdorferi* did not display Tp0954 on their surface, a pattern we previously observed also for Tp0435 lipoprotein (Chan et al., 2016). We confirm that the integrity of outer membrane of spirochetes was maintained during IFA protocol because periplasmic flagella were not labeled with anti-FlaB antibodies followed by anti-mouse Alexa fluor 488 secondary antibodies in either B314(V) or B314(pTp-Bb) spirochetes without permeabilization (Figures 1G,H). The lack of labeling was not due to non-reactivity of antibodies against FlaB protein because both of these B314 derivatives showed strong fluorescence when outer membrane of spirochetes was permeabilized using methanol (green fluorescence in both Figures 1I,J). IFA results showing punctuate labeling on B314 surface using anti-Tp0954N antibodies in another experiment shown in larger version of images further indicates that Tp0954 is indeed a surface protein (Supplementary Figure 3).

### Tp0954-Expressing *B. burgdorferi* B314 Strain Gains the Ability to Bind to Epithelial, Endothelial, Neuronal, and Placental Cell Lines

**Figure 2:**
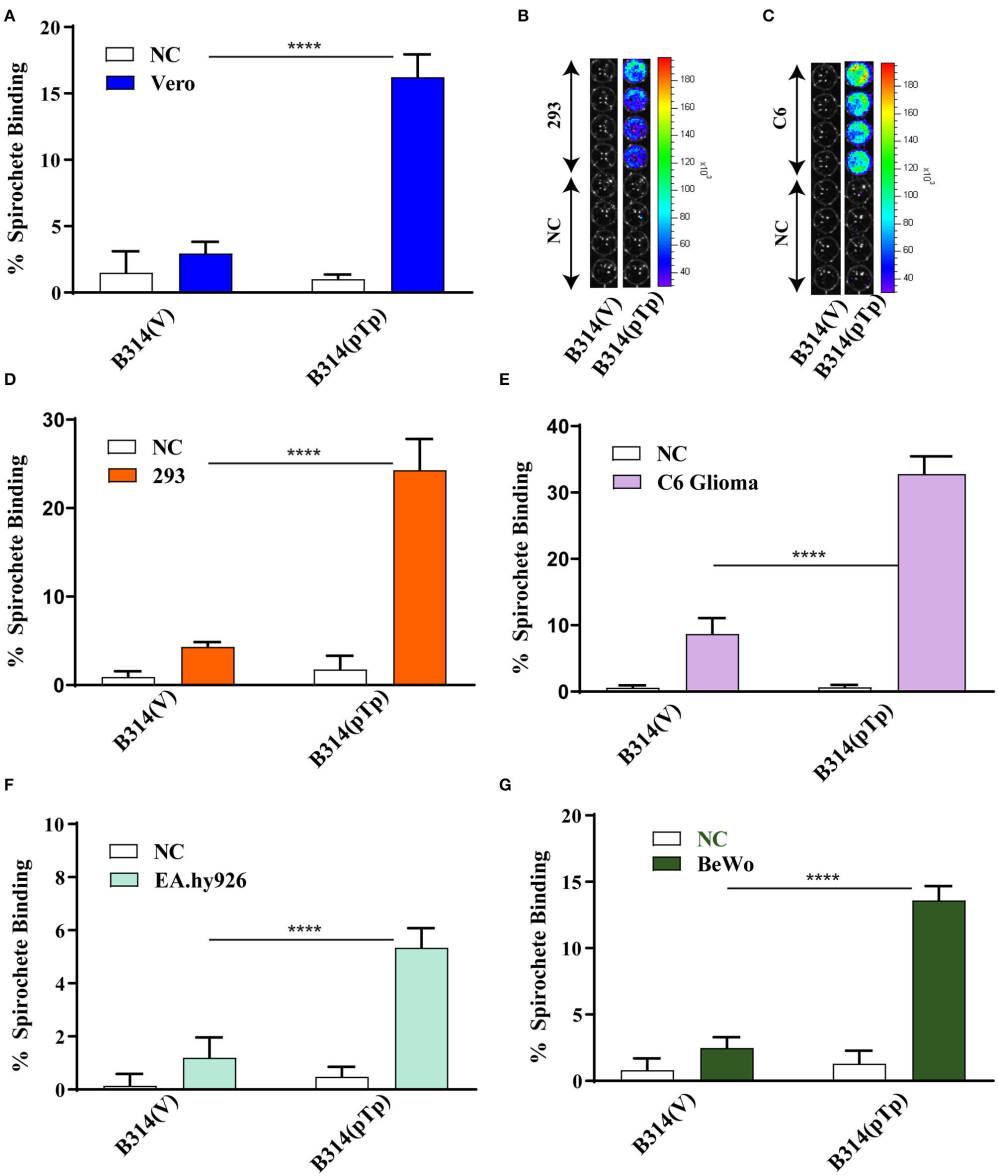
Tp0954-expressing bacteria bind to various mammalian cell lines. **(A,D)** Radiolabeled Tp0954-expressing B314 and plasmid transformed control strains were used to evaluate binding to mammalian epithelial cell lines. B314(pTp-Bb) bound at a 5-fold higher level to Vero cells **(A)**, and 6-fold higher level to HEK293 (293) cells to **(D)** as compared B314 transformed by the empty pJSB175 vector, B314(V). **(B,C)** Binding assay conducted followed by washing and bioluminescence measurement confirmed that B314(pTP-Bb) binds at significantly higher level to 293 and C6 glioma cells. **(E)** B314(pTp-Bb) bound 3.6-fold higher level to the C6 glioma neuronal cells compared to control strain, while **(F)** B314(pTp-Bb) binds relatively weakly to endothelial EA.hy926 cell line. **(G)** Increase in binding of B314(pTp-Bb) was 3.5-fold higher than B314(V) to placental BeWo cell line. Statistically significant difference was determined by unpaired two-tailed *t*-test with Welch's correction (****P < 0.0001).

To determine whether Tp0954 can facilitate attachment to mammalian cell lines, we conducted binding assays using ^35^S- labeled Tp0954-expressing B314 bacteria, B314(pTp-Bb), with B314(V) included as a negative control. We selected cell lines that could be representative of various tissues that are colonized by *T. pallidum* during infection. We show that Tp0954-expressing B314 strain gains an ability to bind to green monkey kidney epithelial Vero cell line at a significantly higher level compared to that in the no-cell (NC) controls. Specifically, B314(pTp-Bb) showed 16% binding to Vero cells (Figure 2A); an ~5-fold increase in binding ability compared to control B314(V) strain, which show only 3% binding to these cells. Additionally, attachment of Tp0954-transformed B314 strain to human embryonic kidney epithelial 293 cells could be confirmed by bioluminescence measurement of the bound spirochetes using *in vivo* Imaging System-50 (IVIS-50) after washing (Figure 2B). Light emission was not observed for B314(V) strain indicating that this control strain does not bind to this cell line to an appreciate level either (Figure 2B). Furthermore, Tp0954-expressing bacteria showed a 6-fold increase in binding ability to the 293 cells (Figure 2D), with an average binding of 24% compared to 4% for control B314(V) strain.

We next determined whether Tp0954-expressing bacteria can bind to cell types representing other tissues. *T. pallidum* colonizes neuronal tissues that results in neurosyphilis manifestations. Vasculature endothelial cells are important for release of *T. pallidum* from blood used as conduit for spirochetes dissemination to various organs resulting in multisystemic syphilis. Therefore, we also examined binding of transformed B314 to the rat C6 glial cell line representing neuronal tissue, and the human endothelial, EA.hy926 cell line. Binding to C6 cells was also observed using bioluminescent detection of the bound transformed B314 spirochetes (Figure 2C). The Tp0954-expressing spirochetes showed a 3.6-fold increase in binding ability to C6 glial cells compared to control strain (Figure 2E), with an average binding of 33 and 9%, respectively. Gain in binding ability of B314 strain on expression of Tp0954 to EA.hy926 cells was relatively low with an average binding of only 5.3% (Figure 2F). This data suggests that Tp0954 may contribute to *T. pallidum* binding during infection only to particular type of cells.

To determine if Tp0954 could also mediate placental colonization, we examined adherence to the human placental fibroblast, BeWo cell line. This is clinically relevant as *T. pallidum* is very efficient in transplacental transmission to fetus. Placental colonization is critical for vertical transmission of these spirochetes to the growing fetus in pregnant women. Binding of Tp0954-expressing B314 spirochetes to BeWo placental cells also increased significantly; they bound placental cells 3.5-fold more efficiently than control B314(V) strain (Figure 2G), with an average binding of 11 and 3%, respectively. The data from these binding assays suggest that Tp0954 protein can promote *T. pallidum* binding to epithelial, neuronal, and placental tissues.

### Tp0954-Expressing B314 Strain Also Binds to Purified Heparin and Dermatan Sulfate

**Figure 3:**
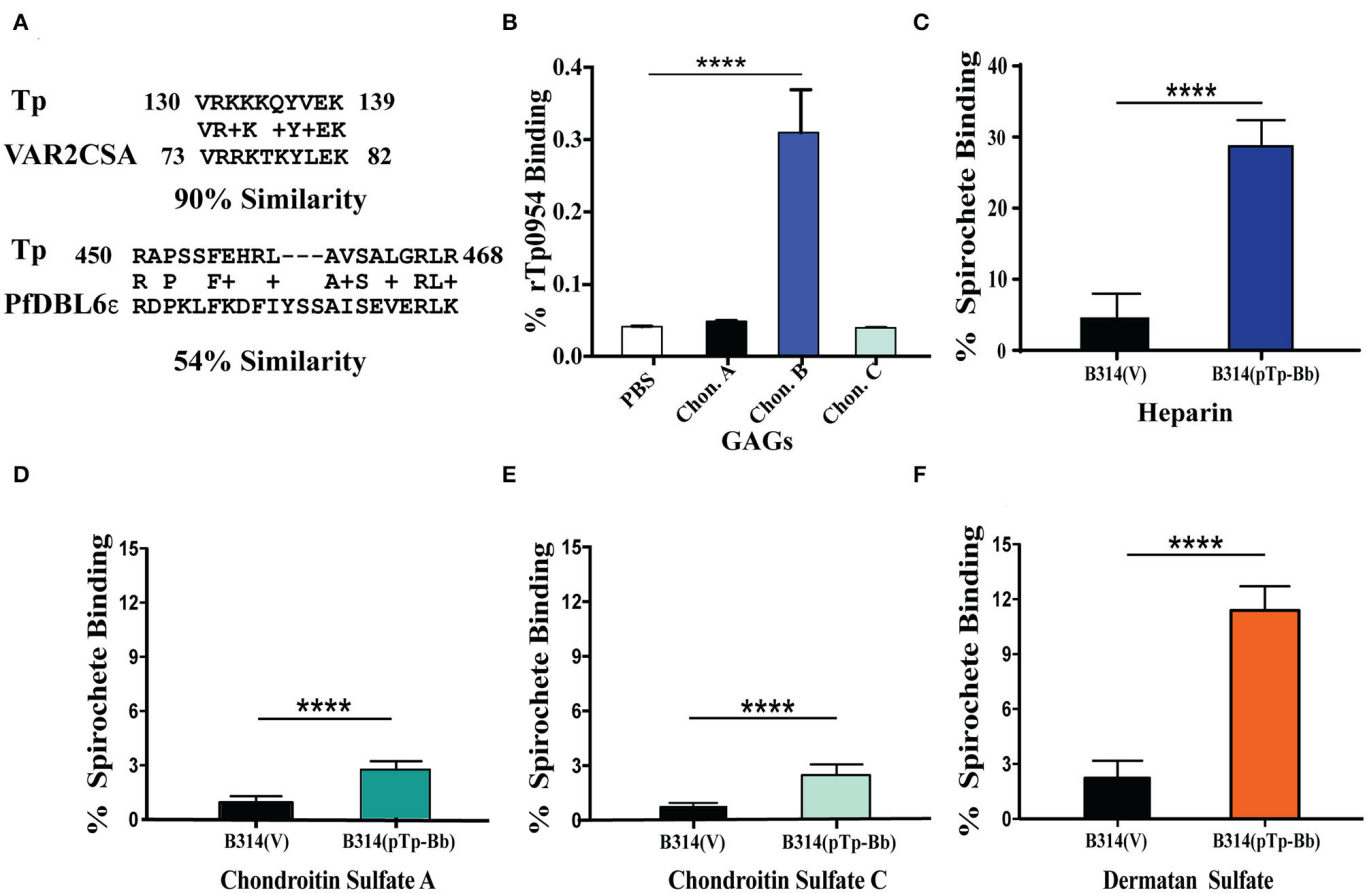
Tp0954 mediated binding to purified GAGs. **(A)** Homology of Tp0954 epitopes with VAR2CSA domains of *P. falciparum* PfEMP1 protein. **(B)** Recombinant Tp0954 protein bound at significantly higher levels to dermatan sulfate than other chondroitin sulfates. **(C)** Binding to heparin, the most negatively charged biological molecule, enhanced in the most pronounced manner on expression of Tp0954 in B314 strain. Tp0954-expressing B314 binding increased minimally to empty wells (PBS) used as negative control (not shown). **(D–F)** Binding of B314(pTp-Bb) increased in the most pronounced manner compared to control B314(V) strain to dermatan sulfate (chondroitin sulfate B) among three types of chondroitin sulfates examined. Statistically significant difference was determined by unpaired two-tailed *t*-test with Welch's correction (****P < 0.0001).

Tp0954-mediated binding to placental cells prompted us to examine homology of this protein to placental binding adhesins of other pathogens. We observed homology between two epitopes of Tp0954 with the sequence of VAR2CSA domain of *P. falciparum* erythrocyte membrane proteins (PfEMP1) that interact with chondroitin sulfate A present on placental cells surface (Figure 3A) and is implicated in congenital malaria (Dahlback et al., 2010; Hviid, 2010; Maestre and Carmona-Fonseca, 2014; Fried and Duffy, 2015). To investigate whether glycosaminoglycans (GAGs) are also involved in the Tp0954 lipoprotein-mediated binding to the mammalian cells, we first examined binding of recombinant Tp0954 proteins to immobilized GAGs on 96-wells plate. Unlike VAR2CSA of *P. falciparum*, Tp0954 bound to dermatan sulfate (chondroitin sulfate B) efficiently (Figure 3B). Since recombinant proteins do not always represent surface epitopes of proteins accurately, we also performed binding assays with the radiolabeled transformed B314 strain to different GAGs. We found that Tp0954-expressing B314 bound to heparin to significantly higher level than control bacteria (Figure 3C). Additionally, Tp0954 expression promotes binding of B314 to dermatan sulfate that is 5-fold higher than the binding levels of control B314(V), and 4-fold higher than binding to closely related GAGs, chondroitin sulfates A and C (compare Figure 3F with Figures 3D,E). Our data suggests that dermatan sulfate and heparin-related GAGs present as components of extracellular matrix (ECM) of host cells may be important receptors recognized by Tp0954 to facilitate binding to various mammalian cells.

**Figure 4:**
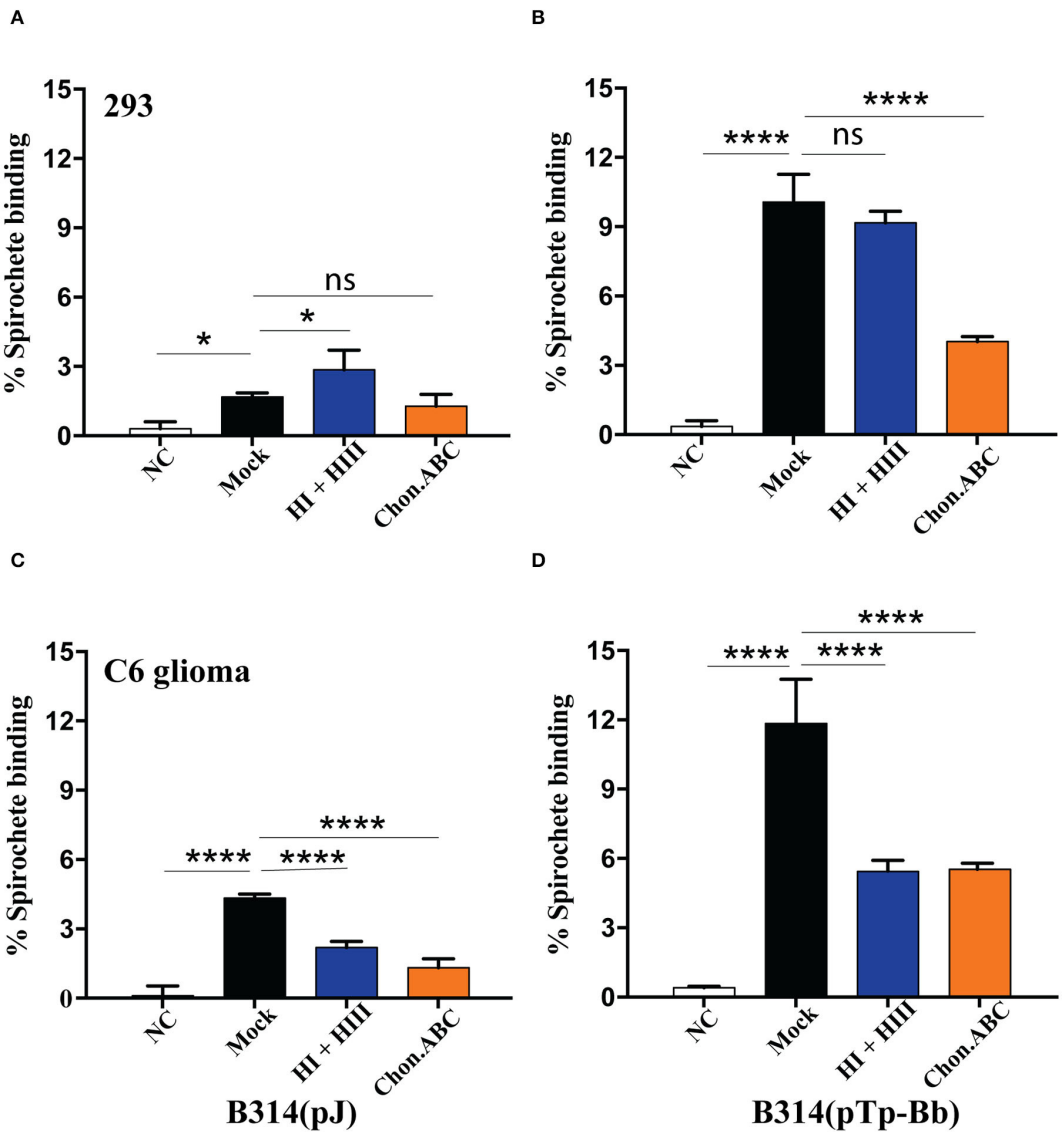
Heparan sulfate and dermatan sulfate as ECM components on specific mammalian cells are required for Tp0954-expressing spirochetes binding. **(A,C)** Low level binding of B314(V) strain were observed to both 293 and C6 glioma cells and removal of GAGs by specific lyases reduced binding further. **(B)** Pretreatment of 293 with chondroitinase ABC and not by a combination of heparinase I and III (HI and HIII) inhibited binding of radiolabeled Tp0954-expressing B314 strain, indicating contribution of dermatan sulfate to adherence on these cells, while **(D)** removal of both heparan sulfate and dermatan sulfate significantly reduced binding of B314(pTp-Bb) to C6 cell line. GAG-lyases are labeled as: NT-No Treatment, Chon-ABC-chondroitinase ABC, and HI+ HII-heparinase I + III. Statistically significant difference was determined by unpaired two-tailed *t*-test with Welch's correction (*P < 0.05, ****P < 0.0001, ns, not significant).

To further confirm the importance of these two GAGs for Tp0954-mediated binding, we used GAG lyases to degrade and remove the specific GAG(s) on cell monolayers before performing binding assays using radiolabeled bacteria. In addition to chondroitin sulfates, the presence of heparan sulfate of human placenta has been shown previously (Isemura et al., 1987; Gabius et al., 1991), often in the immature placenta (Rukosuev, 1992). However, we selected 293 and C6 glioma cell line for this experiment because binding facilitated by Tp0954 was higher on these cells. Again, relatively weak binding of the control B314(V) strain was observed (≤5% binding) to both 293 and C6 glioma cell lines (Figures 4A,C). Degradation of dermatan sulfate using chondroitinase ABC resulted in a significant decrease in the ability of Tp0954-expressing B314 strain to bind to both 293 and C6 glioma cells (Figures 4B,D). Heparan sulfate rather than heparin is present on the surface of mammalian cells. Digestion and removal of heparan sulfate from cell surface by heparinase I and III treatment together did not affect binding to 293 cells significantly. These results agreed with our previous finding that dermatan sulfate is the major GAG present on this cell line (Leong et al., 1998; Parveen et al., 1999). However, removal of heparan sulfate by heparinase I and III on C6 glioma cells resulted in significant reduction of binding of B314(pTp-Bb) to the levels observed after chondroitinase ABC treatment of this cell line (Figure 4D) indicating that both heparan sulfate and dermatan sulfate are recognized by Tp0954 lipoprotein of *T. pallidum* on specific mammalian cells surface. Thus, our results with cell lines demonstrated involvement of heparan sulfate and dermatan sulfate on host cells in interactions with Tp0954 of *T. pallidum*.

### Tp0954 Expression Enhances the Binding of *B. burgdorferi* B314 Strain to Placental Tissue Sections

**Figure 5:**
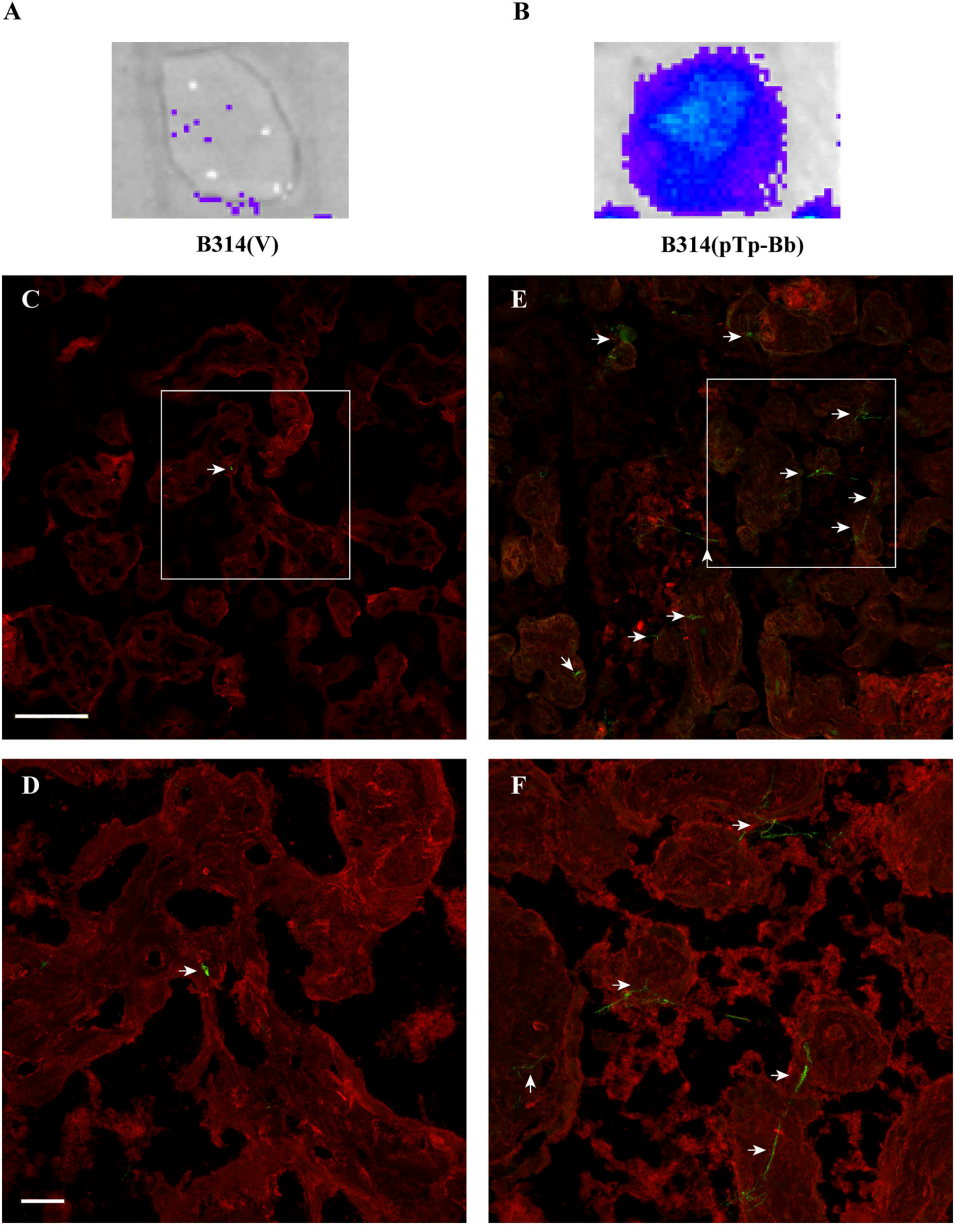
B314 gains ability to bind to human placental tissue sections after Tp0954 expression. **(A,B)** B314(pTp-Bb) strain that expressed Tp0954 on the spirochete surface efficiently binds to placental tissue sections as detected by bioluminescence measurement by IVIS-50 after addition of D-luciferin substrate for luciferase enzyme present in these spirochetes. Control B314(V) strain does not bind to placental tissue significantly confirming poor adherence ability of this strain to mammalian cells. **(C)** A broad overview of binding of control B314(V) labeled with FITC conjugated anti-*B. burgdorferi* antibodies by IFA showed barely detectable spirochetes on placental tissue section that was visualized by labeling with wheat germ agglutinin conjugated Alexa fluor 647 shown by red fluorescence, and **(D)** poor binding by these control spirochetes was confirmed by observation of sections at higher magnification. **(E)** A significant increase in binding to placental tissue section after Tp0954 expression in B314 strain was detected by observation of green fluorescent spirochetes using FITC-conjugated antibodies. **(F)** Spirochetes labeled against *B. burgdorferi* can be more clearly observed at higher magnification depicting significantly higher level of binding of B314(pTp-Bb) compared to B314(V) shown in **(D)**. **(C–F)** Bar on top panels indicate 100 μ and bottom panels depict 20 μ size.

Repeated *in vitro* cultivation of mammalian cell lines sometimes affects expression of surface molecules and receptors. It is possible that BeWo cell line does not fully reflect binding to placental tissues due to some alteration in surface receptors of these transformed cells such that gain in binding was only moderate on these cells when Tp0954 expressing B314 strain was used. Therefore, we conducted binding studies with sections of primary placental tissues obtained from a healthy woman during delivery. To maintain integrity of tissues, we used frozen and fixed placental tissue for sectioning. We first examined binding of our bioluminescent derivatives of B314 strain to frozen and rehydrated human placental tissue sections. Attachment of B314(pTp-Bb) was significantly more pronounced as detected by light emission by bound spirochetes (Figure 5B) as compared to the control B314(V) strain (Figure 5A). We further examined this adherence phenomenon in more details by IFA. In this assay, after allowing spirochetes binding and thorough washing, transformed B314 and placental cells were labeled with fluorescently labeled antibodies against *B. burgdorferi* surface proteins and surface wheat germ agglutinin, respectively. Microscopic images at lower magnification showed a significant increase in binding of green FITC-labeled B314(pTp-Bb) as compared to B314(V) strain (Figures 5C,E). The fields we present here showing <3 bound control spirochetes per microscopic field even at lower magnification. In fact, these images were rarely obtained and the most fields showed no B314(V) control spirochetes bound while binding of B314(pTp-Bb) shown here represent more consistent results throughout sections. Attached spirochetes are more easily discernible at high magnification with a significant increase in binding of B314(pTp-Bb) to placental tissues clearly observed compared to the control B314(V) strain (Figures 5D,F). These results demonstrate that Tp0954 is indeed a placenta binding adhesin. Repetition of the placental binding experiment conducted several times by IFA detected adherence in identical manner. A representative set of images from another experiment are shown (Supplementary Figure 4).

### Tp0954 Is Also Displayed on the Surface of *T. pallidum*

**Figure 6:**
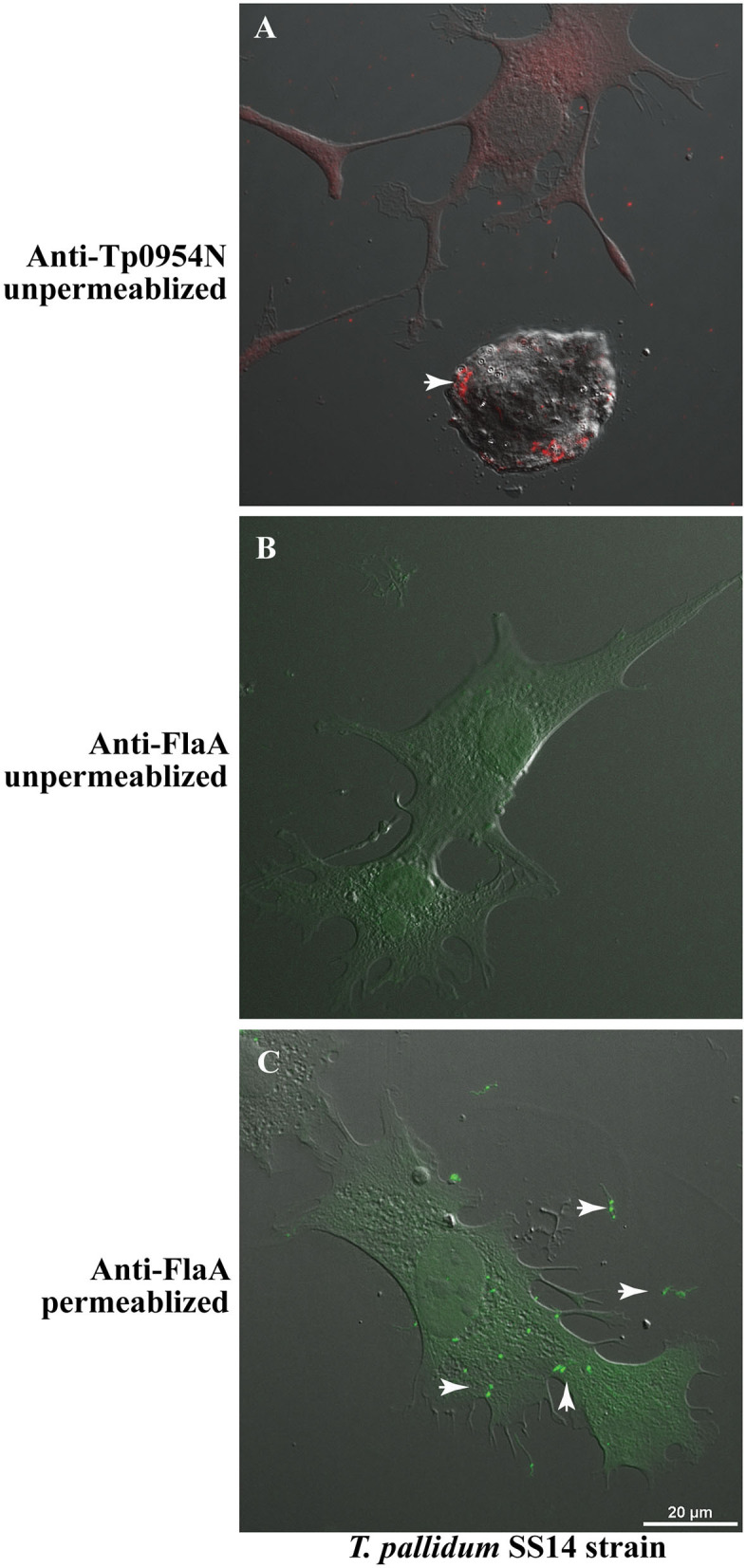
IFA of *T. pallidum* co-cultured with low passage rabbit epithelial Sf1Ep cells. **(A)** IFA using primary polyclonal mouse anti-Tp0954N antibodies followed by TRITC-conjugated secondary antibodies using unpermeabilized *T. pallidum* SS14 strain co-cultured with Sf1Ep cells showed punctate staining confirming the surface localization of Tp0954. **(B)** The lack of staining of flagella by anti-FlaA in unpermeabilized *T. pallidum* indicate that integrity of outer membrane spirochetes was maintained during IFA procedure, and **(C)** staining of flagellar protein after permeabilization indicate antibodies reactivity and periplasmic location of flagella. Bar indicates 20 μ size.

Although the use of a heterologous system is currently the best method to conduct functional analyses of *T. pallidum* proteins in their native state, proof of surface-exposure of *T. pallidum* proteins is considered critical to associate the function of the protein in interaction with the host cells during infection. We examined whether Tp0954 is indeed a surface lipoprotein by conducting IFA using our anti-Tp0954N antibodies with *T. pallidum* SS14 strain grown in co-cultivation with rabbit epithelial Sf1Ep cells *in vitro*. We found that similar to that observed in Tp0954-transformed *B. burgdorferi* B314 strain, this lipoprotein is also displayed on the surface of *T. pallidum* (Figure 6A), thus validating our binding results obtained using the surrogate system. Again, punctuate staining of Tp0954 was observed also on *T. pallidum* surface. We established that integrity of the outer membrane of *T. pallidum* during IFA was retained by including periplasmic flagellar staining as a negative control. The lack of labeling of flagella in unpermeabilized *T. pallidum* with anti-FlaA antibodies (generously provided by Drs. Diane Edmondson and Steven Norris) confirmed that outer membrane was not disrupted in these spirochetes during IFA (Figure 6B). Recognition of flagellar protein by anti-FlaA antibodies was demonstrated by labeling of permeabilized *T. pallidum*. Green fluorescence was detected with these antibodies when labeling was followed by treatment with anti-rabbit Alexa fluor 488 antibodies (Figure 6C). Clear images of fluorescent *T. pallidum* are shown when overlapping DIC of epithelial Sf1Ep cells were not included (Supplementary Figure 5). It is therefore reasonable to assume that Tp0954 present on *T. pallidum* surface also interacts with human cells and bind epithelial, placental, and neuronal cells by recognizing dermatan sulfate and/or heparan sulfate during infection.

## Discussion

The re-emergence of syphilis and increase in congenital syphilis cases is a global health problem that underscores the importance of defining the molecular mechanisms of *T. pallidum* pathogenesis. Although this spirochete was identified as the causative agent of syphilis over a century ago and has been studied ever since, several limitations of working with this pathogen have hindered our understanding of syphilis pathogenesis. Significant knowledge gaps still exist regarding the virulence factors that *T. pallidum* uses to establish infection and persist, subcellular location(s) of these virulence factors, and transcriptional control, just to mention a few topics. Genetic intractability of *T. pallidum* has prevented in-depth studies into its mechanisms of infection. Recently, we developed an effective approach of using heterologous expression system involving a closely related spirochetal species, *B. burgdorferi* to study *T. pallidum* infection. This has led us to make several important discoveries elucidating the mechanisms that facilitate attachment to host components of this obligate extracellular pathogen (Chan et al., 2016; Djokic et al., 2019; Parveen et al., 2019).

In this study, we conducted functional analysis of the *T. pallidum* adhesin Tp0954. Codon-optimization of this protein for expression in *B. burgdorferi* was needed because unlike *T. pallidum, B. burgdorferi* genome is 70% A-T rich, and its preference of degenerate codons is different from that of *T. pallidum* (Fraser et al., 1997, 1998). We also employed the inducible *B. burgdorferi ospC* promoter to allow significant expression of Tp0954 protein at *B. burgdorferi in vitro* culture. Using complementary techniques, we found that codon optimized Tp0954 is well-expressed in *B. burgdorferi*, is processed in a manner similar to that in *T. pallidum* and is localized on the spirochetal surface (Figure 1). Interestingly, multiple isoforms of Tp0954 were detected in both in B314 strain of *B. burgdorferi* and *T. pallidum* Nichols strain by Western blotting suggesting differential post-translational processing similar to that we described for another adhesin, Tp0435 previously (Chan et al., 2016). Limited proteinase K digestion of surface proteins of Tp0954-expressing strain indicate that at least two versions of Tp0954 protein are displayed on the spirochetal surface (Figure 1D). This evidence is in agreement with the predicted surface localization of this lipoprotein (Supplementary Figure 1) and suggests its differential post-translational processing and modification.

Extracellular microaerophilic spirochetal pathogens causing multisystemic diseases employ extensive and often redundant adherence mechanisms to colonize various tissues. We and others have shown previously that, despite its small genome like *T. pallidum*, infectious *B. burgdorferi* possesses multiple redundant molecular adherence mechanisms (Parveen and Leong, 2000; Fischer et al., 2003; Seshu et al., 2006; Weening et al., 2008; Coburn et al., 2013; Schlachter et al., 2018) that are critical for their pathogenesis. The presence of TPR domains in Tp0954 (Jinek et al., 2004) and structural similarity with the PilF crystal structure (2fi7.1A) of *Pseudomonas aeruginosa* (Kim et al., 2006) suggested that Tp0954 could form homodimer or homooligomers or heterooligomers and could be involved in adherence to the host cells. Therefore, we assessed attachment of Tp0954-expressing B314 strain to different cell lines. Binding assays conducted either by measuring radioactivity of labeled spirochetes bound to various cell lines or GAGs by scintillation counter, or by determining bioluminescence of transformed B314 strain of *B. burgdorferi* showed that Tp0954 expression promotes binding of this otherwise poorly adherent strain, as demonstrated by binding of B314(V) strain, to two mammalian epithelial cell lines (Figures 2A,B,D). Moreover, if binding to the specific cell lines is reflection of tissue colonization during infection with *T. pallidum*, binding to other tissue-specific mammalian cell lines, C6 and BeWo, suggests that this adhesin may also play a role in colonization of neuronal and placental tissues that are associated with neurosyphilis and congenital syphilis (Figure 2). Neurosyphilis is a major problem associated with *T. pallidum* infection; however, binding experiments with brain tissue with our transformed B314 strain are not possible because of unavailability of human brain tissue collected and fixed when cells are still alive.

Bacterial adhesins are essential for pathogenesis and disease; consequently, they could be considered as vaccine targets. Although Tp0954 has not been previously studied, it shares limited homology with the VAR2CSA domain of PfEMP1 (Figure 3A), a malarial protein that is displayed on infected red blood cells and facilitates placental colonization of the malaria parasite, *P. falciparum* by recognition of chondroitin sulfate A to cause congenital malaria (Ayres Pereira et al., 2016). In fact, segments of this domain have been tested as vaccine candidates (Fried and Duffy, 2015) specifically targeting congenital malaria. Therefore, we investigated whether specific GAG(s) are also recognized by Tp0954. GAGs are a well-studied class of surface carbohydrates consisting of sugar or uronic acid and hexosamine (glucosamine or galactosamine) disaccharide repeats anchored in the cytoplasmic membrane of host cells to produce proteoglycans that mediate host-pathogen interactions. GAGs are integral components of ECM of mammalian cells. Previous studies have shown the ability of *T. pallidum* adhesins to bind to proteoglycans, fibronectin, and laminin (Brinkman et al., 2008; Kao et al., 2017; Djokic et al., 2019). We show here that unlike VAR2CSA, Tp0954-expressing bacteria bind significantly to purified, immobilized dermatan sulfate and heparin (Figure 3). We further confirmed that the presence of these GAGs contributes to the spirochetal binding to mammalian cells, as removal of heparan sulfate and dermatan sulfate from C6 glioma cell line and of dermatan sulfate from 293 cells resulted in a dramatic reduction of binding by Tp0954-expressing B314 strain (Figure 4). Binding affinities for the specific GAGs on these two cell lines agree with our previous reports using infectious *B. burgdorferi* strains (Leong et al., 1998; Parveen et al., 1999) validating our findings regarding Tp0954-mediated attachment to these cells.

The skin epidermis undergoes constant self-renewal. Proteoglycans as component of ECM are involved in tissue hydration, nutrition, and regulation of cell proliferation and differentiation (Sandjeu and Haftek, 2009). Dermatan sulfate is the predominant GAG in skin cells (Trowbridge and Gallo, 2002) with a small amount of heparan sulfate also present. The central nervous system contains a diverse array of GAGs on different cell types in spatiotemporal manner (Rowlands et al., 2015). GAGs play important biological functions but are also targets for bacterial, viral, and parasitic virulence factors for attachment, invasion, and immune system evasion (Schmidtchen et al., 2001). Heparan sulfate containing proteoglycans have been implicated in neurogenesis, axon guidance, and synapse development (Yamaguchi, 2001). Chondroitin/dermatan sulfate containing proteoglycans are considered important and provide both positive and negative cues during both central and peripheral neuronal development (Hayes et al., 2018). These characteristics of GAGs in these organs offer us insight into how Tp0954 could contribute to colonization of skin causing lesions during primary and secondary syphilis and may also facilitate adherence dependent neurosyphilis manifestation at the late stages of infection when invasion of the brain is common.

GAGs profiles change during placental development (de Oliveira et al., 2015). Unlike young placenta and endometrium that contain predominantly chondroitin sulfate GAGs with smaller amounts of dermatan sulfate and heparan sulfate, human placenta at term contains higher quantities of heparan sulfate and dermatan sulfate. Dermatan sulfate GAGs are associated with fetal blood vessels and syncytial surface. Syndecan primarily contains heparan sulfate predominantly present in syncytiotrophoblast layer while the chondroitin sulfate portion of syndecan-1 are located also in the intervillous space. Syndecan-1 is a dimeric proteoglycan in which the ectodomain includes four binding sites for chondroitin sulfate that are proximal to the transmembrane domain, and six distal binding sites primarily for heparan sulfate. The intervillous space contains syndecan-1 ectodomains free of heparan sulfate, while heparan sulfate is present in trophoblast layer. The presence of heparan sulfate proteoglycans in the oviduct, in the uterus and in human follicular fluid, suggests that they could play additional distal roles during gestation. Decorin is a small leucine-rich proteoglycan produced by stromal and other cells of the pregnant endometrium (Lala and Nandi, 2016). Decorin and biglycan contain chondroitin and dermatan sulfates. Decorin is present in the stroma and surrounds the fetal blood vessels of the villous placenta while biglycan lies in the endothelium and in the smooth muscular cells of the fetal capillaries. Overall, these reports and our findings here suggest that heparan sulfates could facilitate binding of *T. pallidum* on the maternal side of the placenta while dermatan sulfate could promote colonization of vasculature on the fetus side of the placenta. Significance of these colonization mechanisms underline the highly efficient transplacental transmission of this spirochete from mother to child resulting in congenital syphilis and adverse pregnancy outcomes.

## Summary

Tp0954 was selected for this study because based upon our bioinformatics analysis it was expected to be a surface exposed lipoprotein of *T. pallidum* and a potential virulence factor. We demonstrate here that Tp0954 is indeed a surface-exposed adhesin that mediates binding of spirochetes to the host epithelial, neuronal, and placental cells. This work presents the first evidence of a *T. pallidum* protein importance for placental colonization and potentially transplacental transmission from mother to fetus during pregnancy to cause congenital syphilis. While previous protective immunity investigations have yielded limited success (Pavia et al., 1985; Wicher et al., 1992), further studies are necessary here to identify more promising vaccine candidates. Our study here lays foundation to determine whether Tp0954 could be an effective vaccine candidate to prevent mother to child transmission in resource poor regions of the world where prenatal care and hence, treatment of pregnant women in timely manner does not occur resulting in a high number of cases of congenital syphilis. To assess this possibility, we would like to determine in the future whether infected pregnant women with high titer of antibodies against Tp0954 have favorable pregnancy outcome. Consistent results from several patients will help us determine whether Tp0954 is indeed a potentially useful vaccine candidate. Such results will suggest that prevention of transplacental mother to child transmission could be facilitated by Tp0954-based vaccine when used alone, or with other protective antigenic candidates to prevent vertical transmission of *T. pallidum*, inhibiting congenital syphilis particularly in countries with limited resources to reach different regions for prenatal care.

## Data Availability Statement

The original contributions presented in the study are included in the article/Supplementary Materials, further inquiries can be directed to the corresponding author.

## Ethics Statement

The animal study was reviewed and approved by Rutgers Biomedical and Health Sciences IACUC.

## Author Contributions

NP designed the study, supervised different aspects of the project and conducted IFA, and placental binding experiment. SP cloned *tp0954* gene, confirmed its expression in B314 strain, and conducted majority of binding experiments. SCR determined the role of GAGs in binding of this strain to 293 and C6 glioma cells. Co-culture of *T. pallidum* was conducted under LG's supervision. All authors contributed to the article and approved the submitted version.

## Conflict of Interest

The authors declare that the research was conducted in the absence of any commercial or financial relationships that could be construed as a potential conflict of interest.

## Supplementary Material

Supplementary Figure 1 Prediction of Tp0954 as surface lipoprotein of *T. pallidum* and modeling by Swiss Model. **(A)** First 19 amino acids marked as transmembrane peptide depict the signal peptide that likely anchors the protein in the cytoplasmic membrane and is cleaved in mature lipoprotein. The lipoprotein is predicted to be inserted in the outer membrane by lipid moiety at N-terminal end and peptide at around 350 amino acid residues site of the ORF while most of the Tp0954 lipoprotein, particularly N-terminal region, is predicted to be on the outer surface of the spirochetes. **(B)** Sequence prediction of Tp0954 using LipoP 1.0 indicates that Tp0954 is likely a lipoprotein in which the lipobox LFVSC is predicted by SpLip software with 20th amino acid, a cysteine is expected to be the first amino acid of the mature protein. **(C)** The SWISS-MODEL template library (SMTL version 2017-09-21, PDB release 2017-09-15) was searched with Blast and HHBlits for evolutionary related structures matching the target Tp0954 sequence. Predicted structure is shown.

Supplementary Figure 2 Tp0954 is a highly conserved protein of *T. pallidum* that also shows high homology with *T. paraluiscuniculi* protein. **(A)** Sequence homology of Tp0954 from five *T. pallidum* sequenced genomes originated in different regions of the world. Aligned sequences of Tp0954 proteins from *T. pallidum* strains Nichols (Accession No. AAC65909), SS14 (Accession No. AGN77095.1), Chicago (Accession No. ADD73044.1), Mexico A (Accession No. AFU66929.1), and Amoy from China (Accession No. ANA42627.1) show perfect identity. **(B)** Alignment of Tp0954 from *T. pallidum* Nichols Houston strain (Accession No. ANI45721.1) and of *T. paraluiscuniculi* strain (Accession No. WP_013945516.1) shows almost perfectly identical sequences.

Supplementary Figure 3 Surface labeling detected only in Tp0954 transformed B314 strain and flagella staining observed only after spirochetes permeabilization. **(A,B)** IFA using primary antibodies against 281 N-terminal amino acids of mature Tp0954 protein followed by TRITC-labeled secondary antibodies using unpermeabilized spirochetes confirmed the surface localization of Tp0954 only in B314(pTp-Bb) and not in the control, B314(V) strain. **(C)** The lack of staining of flagella in unpermeabilized Tp0954 transformed B314 spirochetes by anti-FlaB monoclonal antibodies treatment followed by anti-mouse Alexa fluor 488 indicate that integrity of outer membrane of B314 was maintained during IFA procedure. **(D)** Staining of spirochetes after permeabilization with methanol detected green *B. burgdorferi* periplasmic flagella depicting the specific reactivity of anti-FlaB antibodies. Spirochetes DNA were co-labeled with DAPI and examined in parallel (not shown). Bar indicates 20 μ size.

Supplementary Figure 4 B314 strain gains ability to bind to placental tissue section on expression of Tp0954 of *T. pallidum*. In this second experiment, **(A)** overview of binding of control B314(V) could be barely detected on placental tissue section by IFA, and **(B)** was confirmed by observation of sections at higher magnification. **(C)** A significant increase in binding after Tp0954 expression in B314 strain was observed by detection of green fluorescent spirochetes attached to placental cells, which were labeled with wheat germ agglutinin conjugated to Alexa fluor 647 as shown by red fluorescence, compared to control B314(V) strain **(A,B)**. **(D)** Spirochetes labeled by FITC-conjugated antibodies against *B. burgdorferi* can be more clearly observed at higher magnification with significantly higher level of binding of B314(pTp-Bb) than B314(V) in **(B)**. **(A,C)** Bar on top panels indicate 100 μ while **(B,D)** bottom panels depict 20 μ size.

Supplementary Figure 5 Labeling of Tp0954 on the surface and flagella in the periplasmic space of *T. pallidum* SS14 strain. **(A)** IFA using primary polyclonal mouse anti-Tp0954N antibodies followed by TRITC-conjugated secondary antibodies using unpermeabilized *T. pallidum* SS14 strain co-cultured with Sf1Ep cells showed punctate staining confirming the surface localization of Tp0954. **(B)** The lack of staining of flagella in unpermeabilized *T. pallidum* indicate that integrity of outer membrane spirochetes was maintained during IFA procedure, and **(C)** staining after permeabilization confirm periplasmic location of flagella. Data using TRITC filter **(A)**, and FITC filter **(B,C)** are shown. DIC images to show mammalian cells are not included in the figure for clarity. Bar indicates 20 μ size.
