# Supplementary material for: Identification and Functional Assessment of the First Placental Adhesin of Treponema pallidum That May Play Critical Role in Congenital Syphilis
Source: Front Microbiol. 2020 Dec 21;11:621654. doi: 10.3389/fmicb.2020.621654 (PMC7779807; doi:10.3389/fmicb.2020.621654)
Supplement: Supplementary Figure 1 — Prediction of Tp0954 as surface lipoprotein of T. pallidum and modeling by Swiss Model. (A) First 19 amino acids marked as transmembrane peptide depict the signal peptide that likely anchors the protein in the cytoplasmic membrane and is cleaved in mature lipoprotein. The lipoprotein is predicted to be inserted in the outer membrane by lipid moiety at N-terminal end and peptide at around 350 amino acid residues site of the ORF while most of the Tp0954 lipoprotein, particularly N-terminal region, is predicted to be on the outer surface of the spirochetes. (B) Sequence prediction of Tp0954 using LipoP 1.0 indicates that Tp0954 is likely a lipoprotein in which the lipobox LFVSC is predicted by SpLip software with 20th amino acid, a cysteine is expected to be the first amino acid of the mature protein. (C) The SWISS-MODEL template library (SMTL version 2017-09-21, PDB release 2017-09-15) was searched with Blast and HHBlits for evolutionary related structures matching the target Tp0954 sequence. Predicted structure is shown. [file Image_1.pdf]

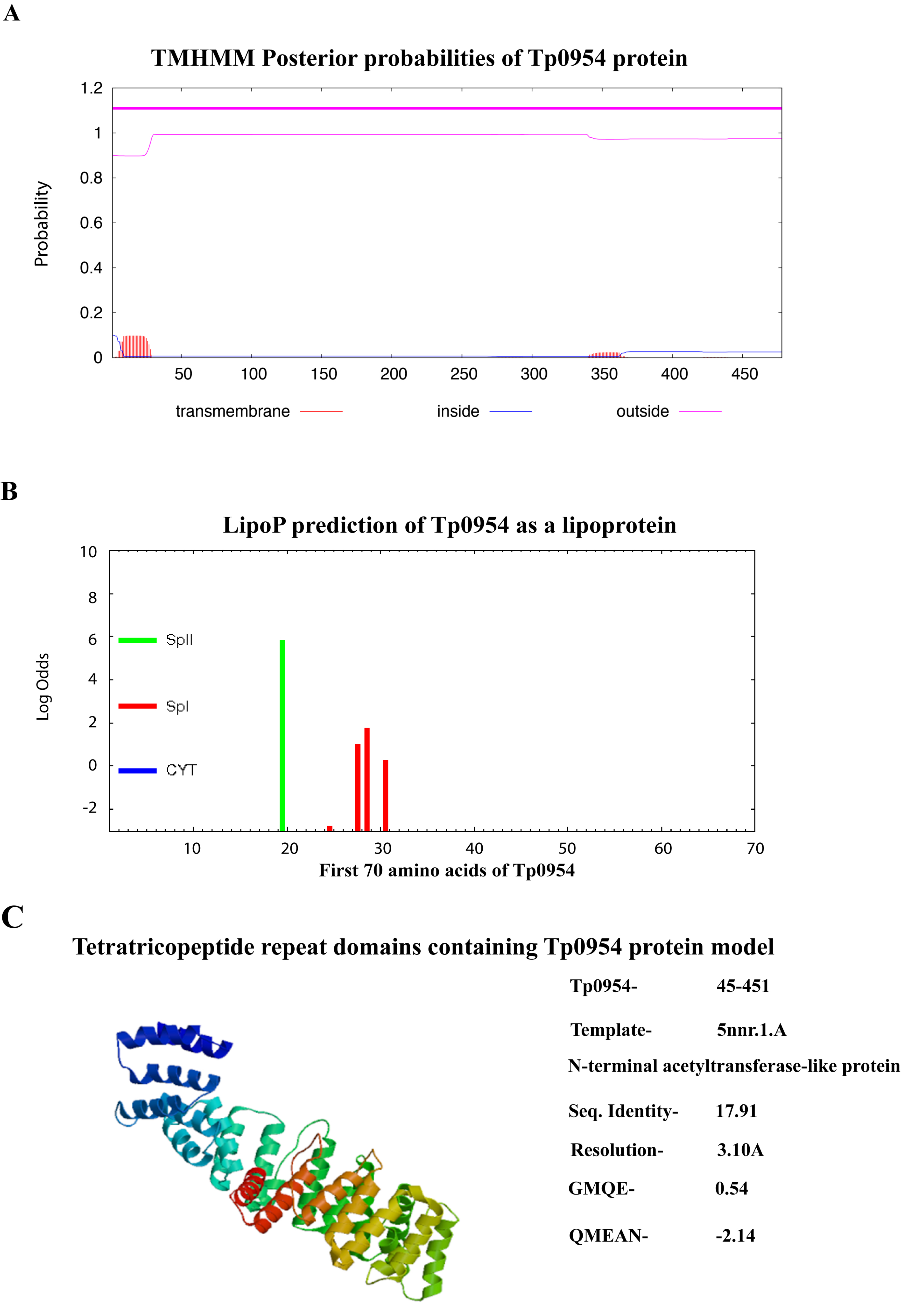

A
TMHMM Posterior probabilities of Tp0954 protein
Probability
1.2
1
0.8
0.6
0.4
0.2
0
50
100
150
200
250
300
350
400
450
transmembrane
inside
outside
B
LipoP prediction of Tp0954 as a lipoprotein
Log Odds
10
8
6
4
2
0
-2
SpII
SpI
CYT
10
20
30
40
50
60
70
First 70 amino acids of Tp0954
C
Tetratricopeptide repeat domains containing Tp0954 protein model
Tp0954- 45-451
Template- 5nnr.1.A
N-terminal acetyltransferase-like protein
Seq. Identity- 17.91
Resolution- 3.10A
GMQE- 0.54
QMEAN- -2.14
