# Supplementary material for: Identification and Functional Assessment of the First Placental Adhesin of Treponema pallidum That May Play Critical Role in Congenital Syphilis
Source: Front Microbiol. 2020 Dec 21;11:621654. doi: 10.3389/fmicb.2020.621654 (PMC7779807; doi:10.3389/fmicb.2020.621654)
Supplement: Supplementary Figure 2 — Tp0954 is a highly conserved protein of T. pallidum that also shows high homology with T. paraluiscuniculi protein. (A) Sequence homology of Tp0954 from five T. pallidum sequenced genomes originated in different regions of the world. Aligned sequences of Tp0954 proteins from T. pallidum strains Nichols (Accession No. AAC65909), SS14 (Accession No. AGN77095.1), Chicago (Accession No. ADD73044.1), Mexico A (Accession No. AFU66929.1), and Amoy from China (Accession No. ANA42627.1) show perfect identity. (B) Alignment of Tp0954 from T. pallidum Nichols Houston strain (Accession No. ANI45721.1) and of T. paraluiscuniculi strain (Accession No. WP_013945516.1) shows almost perfectly identical sequences. [file Image_2.pdf]

## A. Alignment of Tp0954 sequence from five different T. pallidum strain of different geographic origins

```
Nichols    1 MRYGTLFKVSVLLGALFVSCVSTGSNSARESERAQLLKSENPNIRFAAQLSGLLEKQRWEEALQLFDTLSPEHRAEKRIQYLYLSTLISAGKLTHAQELA
SS14       1 MRYGTLFKVSVLLGALFVSCVSTGSNSARESERAQLLKSENPNIRFAAQLSGLLEKQRWEEALQLFDTLSPEHRAEKRIQYLYLSTLISAGKLTHAQELA
Chicago    1 MRYGTLFKVSVLLGALFVSCVSTGSNSARESERAQLLKSENPNIRFAAQLSGLLEKQRWEEALQLFDTLSPEHRAEKRIQYLYLSTLISAGKLTHAQELA
Mexico A   1 MRYGTLFKVSVLLGALFVSCVSTGSNSARESERAQLLKSENPNIRFAAQLSGLLEKQRWEEALQLFDTLSPEHRAEKRIQYLYLSTLISAGKLTHAQELA
Amoy       1 MRYGTLFKVSVLLGALFVSCVSTGSNSARESERAQLLKSENPNIRFAAQLSGLLEKQRWEEALQLFDTLSPEHRAEKRIQYLYLSTLISAGKLTHAQELA

             ****************************************************************************************************

Nichols      EKLGQDGPTASETVQLWYAHAMIAQAKRDVRKKKQYVEKILAQDPHDLWALTERGYDFLSVNDYAQAVQAFSRALRVEPRAQDARVGLGKVYYLQGKMQE
SS14         EKLGQDGPTASETVQLWYAHAMIAQAKRDVRKKKQYVEKILAQDPHDLWALTERGYDFLSVNDYAQAVQAFSRALRVEPRAQDARVGLGKVYYLQGKMQE
Chicago      EKLGQDGPTASETVQLWYAHAMIAQAKRDVRKKKQYVEKILAQDPHDLWALTERGYDFLSVNDYAQAVQAFSRALRVEPRAQDARVGLGKVYYLQGKMQE
Mexico A     EKLGQDGPTASETVQLWYAHAMIAQAKRDVRKKKQYVEKILAQDPHDLWALTERGYDFLSVNDYAQAVQAFSRALRVEPRAQDARVGLGKVYYLQGKMQE
Amoy         EKLGQDGPTASETVQLWYAHAMIAQAKRDVRKKKQYVEKILAQDPHDLWALTERGYDFLSVNDYAQAVQAFSRALRVEPRAQDARVGLGKVYYLQGKMQE

             ****************************************************************************************************

Nichols      AEAQYRQVLQDTPEHERALAECARVKAETNRVLEAIRDLERVVQLDPHDPAYWTDLGTYLSQAGKKERAAAAFERAVALSADAYFAHIYLGGIYDELGRA
SS14         AEAQYRQVLQDTPEHERALAECARVKAETNRVLEAIRDLERVVQLDPHDPAYWTDLGTYLSQAGKKERAAAAFERAVALSADAYFAHIYLGGIYDELGRA
Chicago      AEAQYRQVLQDTPEHERALAECARVKAETNRVLEAIRDLERVVQLDPHDPAYWTDLGTYLSQAGKKERAAAAFERAVALSADAYFAHIYLGGIYDELGRA
Mexico A     AEAQYRQVLQDTPEHERALAECARVKAETNRVLEAIRDLERVVQLDPHDPAYWTDLGTYLSQAGKKERAAAAFERAVALSADAYFAHIYLGGIYDELGRA
Amoy         AEAQYRQVLQDTPEHERALAECARVKAETNRVLEAIRDLERVVQLDPHDPAYWTDLGTYLSQAGKKERAAAAFERAVALSADAYFAHIYLGGIYDELGRA

             ****************************************************************************************************

Nichols      EKAIEHYQRAVQLYPKYHFSFESLGVLFWEQQRWEEAREAFATALTYAPTNISYALMTALCLCQMGQAHKAQHFMRTFIRTVDRTRREVEYFLCRLFVDL
SS14         EKAIEHYQRAVQLYPKYHFSFESLGVLFWEQQRWEEAREAFATALTYAPTNISYALMTALCLCQMGQAHKAQHFMRTFIRTVDRTRREVEYFLCRLFVDL
Chicago      EKAIEHYQRAVQLYPKYHFSFESLGVLFWEQQRWEEAREAFATALTYAPTNISYALMTALCLCQMGQAHKAQHFMRTFIRTVDRTRREVEYFLCRLFVDL
Mexico A     EKAIEHYQRAVQLYPKYHFSFESLGVLFWEQQRWEEAREAFATALTYAPTNISYALMTALCLCQMGQAHKAQHFMRTFIRTVDRTRREVEYFLCRLFVDL
Amoy         EKAIEHYQRAVQLYPKYHFSFESLGVLFWEQQRWEEAREAFATALTYAPTNISYALMTALCLCQMGQAHKAQHFMRTFIRTVDRTRREVEYFLCRLFVDL

             ****************************************************************************************************

Nichols      SGEQDMASRISKIKSVPQRIRYSFYLAFFYELGGRHLLAEKHYGEVESARAPSSFEHRLAVSALGRLRGRSSSLRTNP 478
SS14         SGEQDMASRISKIKSVPQRIRYSFYLAFFYELGGRHLLAEKHYGEVESARAPSSFEHRLAVSALGRLRGRSSSLRTNP 478
Chicago      SGEQDMASRISKIKSVPQRIRYSFYLAFFYELGGRHLLAEKHYGEVESARAPSSFEHRLAVSALGRLRGRSSSLRTNP 478
Mexico A     SGEQDMASRISKIKSVPQRIRYSFYLAFFYELGGRHLLAEKHYGEVESARAPSSFEHRLAVSALGRLRGRSSSLRTNP 478
Amoy         SGEQDMASRISKIKSVPQRIRYSFYLAFFYELGGRHLLAEKHYGEVESARAPSSFEHRLAVSALGRLRGRSSSLRTNP 478
             ******************************************************************************
```

## B. Alignment of Tp0954 of Nichols Houston and *T. paraluiscuniculi* homolog

```
Houston      MRYGTLFKVSVLLGALFVSCVSTGSNSARESERAQLLKSENPNIRFAAQLSGLLEKQRWEEALQLFDTLSPEHRAEKRIQYLYLSTLISAGKLTHAQELA
Paraluisc.   MRYGTLFKVSVLLGALFVSCVSTGSNSARESERAQLLKSENPNIRFAAQLSGLLEKQRWEEALQLFDTLSPEHRAEKRIQYLYLSTLISAGKLTHAQELA

             ****************************************************************************************************

Houston      EKLGQDGPTASETVQLWYAHAMIAQAKRDVRKKKQYVEKILAQDPHDLWALTERGYDFLSVNDYAQAVQAFSRALRVEPRAQDARVGLGKVYYLQGKMQE
Paraluisc.   EKLGQDGPTASETVQLWYAHAMIAQAKRDVRKKKQYVEKILAQDPHDLWALTERGYDFLSVNDYAQAVQAFSRALRVEPRAQDARVGLGKVYYLQGKMQE

             ****************************************************************************************************

Houston      AEAQYRQVLQDTPEHERALAECARVKAETNRVLEAIRDLERVVQLDPHDPAYWTDLGTYLSQAGKKERAAAAFERAVALSADAYFAHIYLGGIYDELGRA
Paraluisc.   AEAQYRQVLQDTPEHERALAECARVKAETNRVLEAIRDLERVVQLDPHDPAYWTDLGTYLSQAGKKERAAAAFERAVALSADAYFAHIYLGGIYDELGRA

             ****************************************************************************************************

Houston      EKAIEHYQRAVQLYPKYHFSFESLGVLFWEQQRWEEAREAFATALTYAPTNISYALMTALCLCQMGQAHKAQHFMRTFIRTVDRTRREVEYFLCRLFVDL
Paraluisc.   EKAIEHYQRAVQLYPKYHFSFESLGVLFWEQQRWEEAREAFATALTYAPTNISYALMTALCLCQMGQAHKAQHFMRTFIRTVDRTRREVEYFLCRLFVDL

             ****************************************************************************************************

Houston      SGEQDMASRISKIKSVPQRIRYSFYLAFFYELGGRHLLAEKHYGEVESARAPSSFEHRLAVSALGRLRGRSSSLRTNP 478
Paraluisc.   SGEQDMASRISKIKSVPQRIRYSFYLAFFYELGGRHLLAEKHYGEVESARAPSSFEHRLAVSALGRLRRRSSSLRTNP 478
             ******************************************************************** *********
```
