# Supplementary figures and images for: Identification and Functional Assessment of the First Placental Adhesin of Treponema pallidum That May Play Critical Role in Congenital Syphilis
Source: Front Microbiol. 2020 Dec 21;11:621654. doi: 10.3389/fmicb.2020.621654 (PMC7779807; doi:10.3389/fmicb.2020.621654)

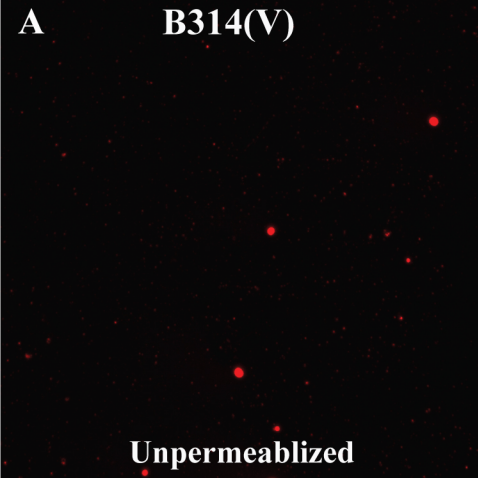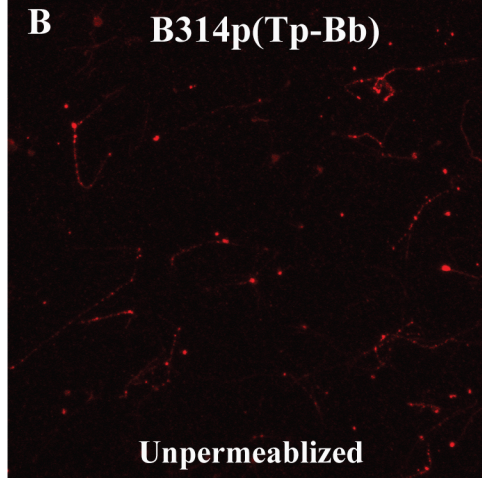

**Anti-Tp954N**

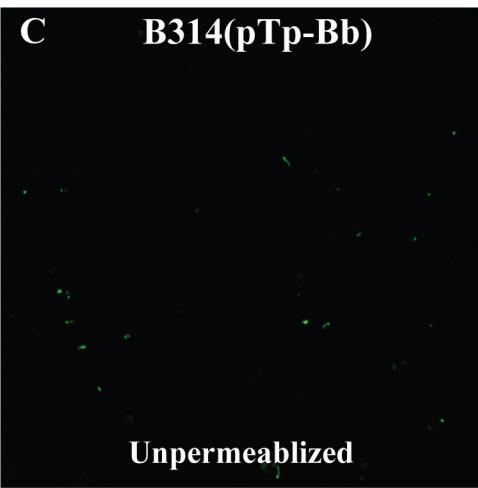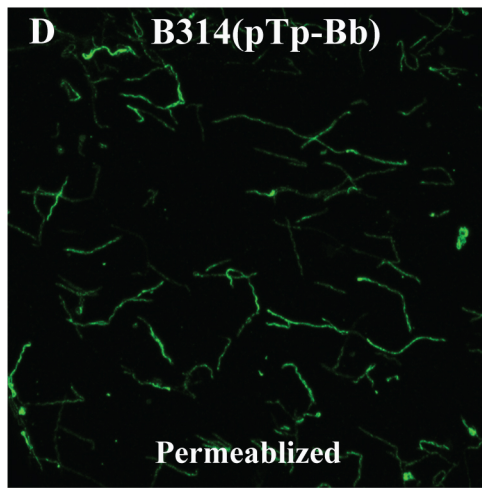

**Anti-FlaA**

Supplement: Supplementary Figure 3 — Surface labeling detected only in Tp0954 transformed B314 strain and flagella staining observed only after spirochetes permeabilization. (A,B) IFA using primary antibodies against 281 N-terminal amino acids of mature Tp0954 protein followed by TRITC-labeled secondary antibodies using unpermeabilized spirochetes confirmed the surface localization of Tp0954 only in B314(pTp-Bb) and not in the control, B314(V) strain. (C) The lack of staining of flagella in unpermeabilized Tp0954 transformed B314 spirochetes by anti-FlaB monoclonal antibodies treatment followed by anti-mouse Alexa fluor 488 indicate that integrity of outer membrane of B314 was maintained during IFA procedure. (D) Staining of spirochetes after permeabilization with methanol detected green B. burgdorferi periplasmic flagella depicting the specific reactivity of anti-FlaB antibodies. Spirochetes DNA were co-labeled with DAPI and examined in parallel (not shown). Bar indicates 20 μ size. [file Image_3.pdf]

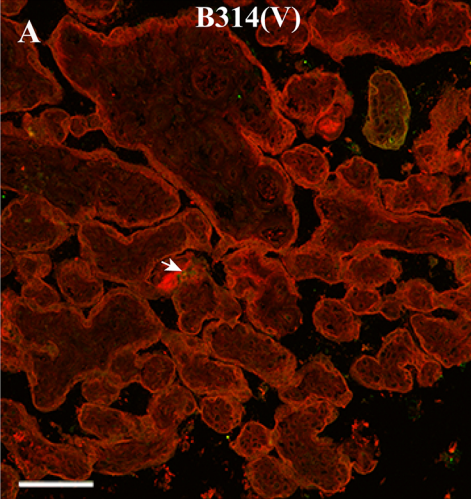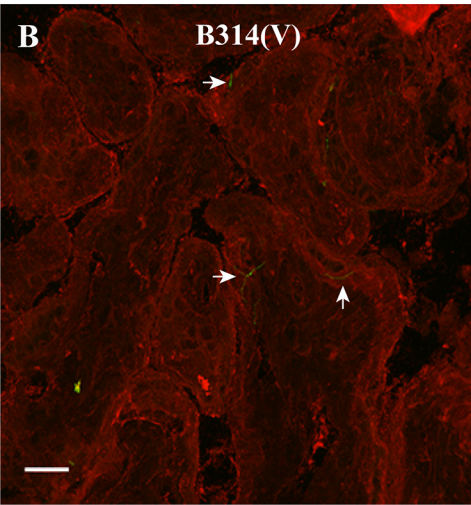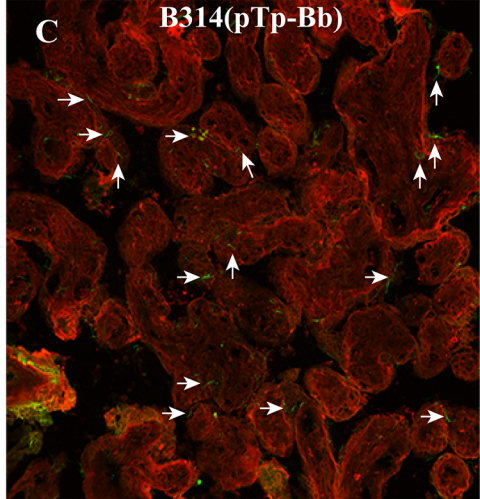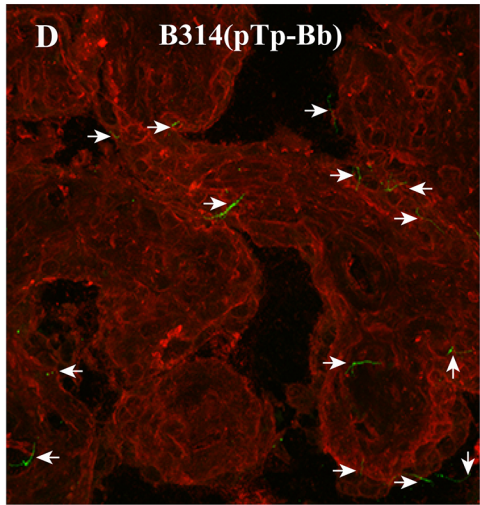

Supplement: Supplementary Figure 4 — B314 strain gains ability to bind to placental tissue section on expression of Tp0954 of T. pallidum. In this second experiment, (A) overview of binding of control B314(V) could be barely detected on placental tissue section by IFA, and (B) was confirmed by observation of sections at higher magnification. (C) A significant increase in binding after Tp0954 expression in B314 strain was observed by detection of green fluorescent spirochetes attached to placental cells, which were labeled with wheat germ agglutinin conjugated to Alexa fluor 647 as shown by red fluorescence, compared to control B314(V) strain (A,B). (D) Spirochetes labeled by FITC-conjugated antibodies against B. burgdorferi can be more clearly observed at higher magnification with significantly higher level of binding of B314(pTp-Bb) than B314(V) in (B). (A,C) Bar on top panels indicate 100 μ while (B,D) bottom panels depict 20 μ size. [file Image_4.pdf]
