# Supplementary material for: Identification and Functional Assessment of the First Placental Adhesin of Treponema pallidum That May Play Critical Role in Congenital Syphilis
Source: Front Microbiol. 2020 Dec 21;11:621654. doi: 10.3389/fmicb.2020.621654 (PMC7779807; doi:10.3389/fmicb.2020.621654)
Supplement: Supplementary Figure 5 — Labeling of Tp0954 on the surface and flagella in the periplasmic space of T. pallidum SS14 strain. (A) IFA using primary polyclonal mouse anti-Tp0954N antibodies followed by TRITC-conjugated secondary antibodies using unpermeabilized T. pallidum SS14 strain co-cultured with Sf1Ep cells showed punctate staining confirming the surface localization of Tp0954. (B) The lack of staining of flagella in unpermeabilized T. pallidum indicate that integrity of outer membrane spirochetes was maintained during IFA procedure, and (C) staining after permeabilization confirm periplasmic location of flagella. Data using TRITC filter (A), and FITC filter (B,C) are shown. DIC images to show mammalian cells are not included in the figure for clarity. Bar indicates 20 μ size. [file Image_5.pdf]

Anti-Tp954N
unpermeablized

Anti-FlaA
unpermeablized

Anti-FlaA
permeablized

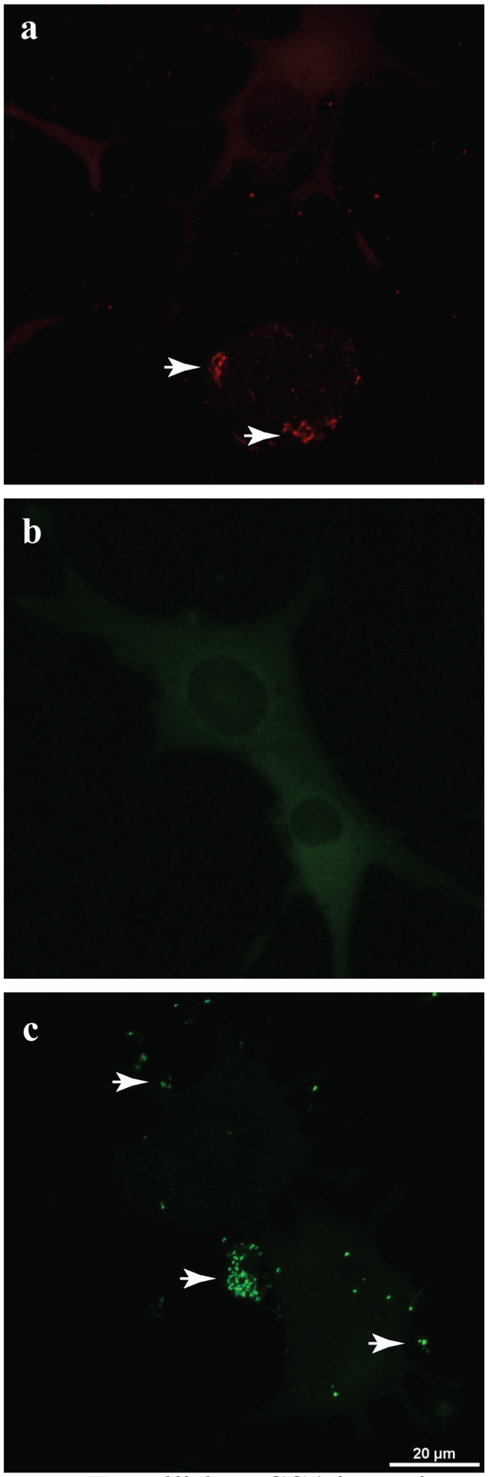

T. pallidum SS14 strain
